# Nanoplastics Detected in Commercial Sea Salt

**DOI:** 10.1021/acs.est.3c11021

**Published:** 2024-05-06

**Authors:** Xuejun Ruan, Jianpeng Ao, Minglu Ma, Robin R. Jones, Juan Liu, Kejian Li, Qiuyue Ge, Guanjun Xu, Yangyang Liu, Tao Wang, Lifang Xie, Wei Wang, Wenbo You, Licheng Wang, Ventsislav K. Valev, Minbiao Ji, Liwu Zhang

**Affiliations:** † Shanghai Key Laboratory of Atmospheric Particle Pollution and Prevention, National Observations and Research Station for Wetland Ecosystems of the Yangtze Estuary, IRDR International Center of Excellence on Risk Interconnectivity and Governance on Weather, Department of Environmental Science & Engineering, Fudan University, Shanghai 200433, Peoples’ Republic of China; ‡ State Key Laboratory of Surface Physics and Department of Physics, Human Phenome Institute, Academy for Engineering and Technology, Key Laboratory of Micro and Nano Photonic Structures (Ministry of Education), Yiwu Research Institute of Fudan University, Fudan University, Shanghai 200433, Peoples’ Republic of China; § Centre for Photonics and Photonic Materials and Centre for Nanoscience and Nanotechnology, Department of Physics, University of Bath, Claverton Down, Bath BA2 7AY, U.K.; ∥ Shanghai Institute of Pollution Control and Ecological Security, Shanghai 200092, Peoples’ Republic of China

**Keywords:** nanoplastics, sea salt, SERS, SRS

## Abstract

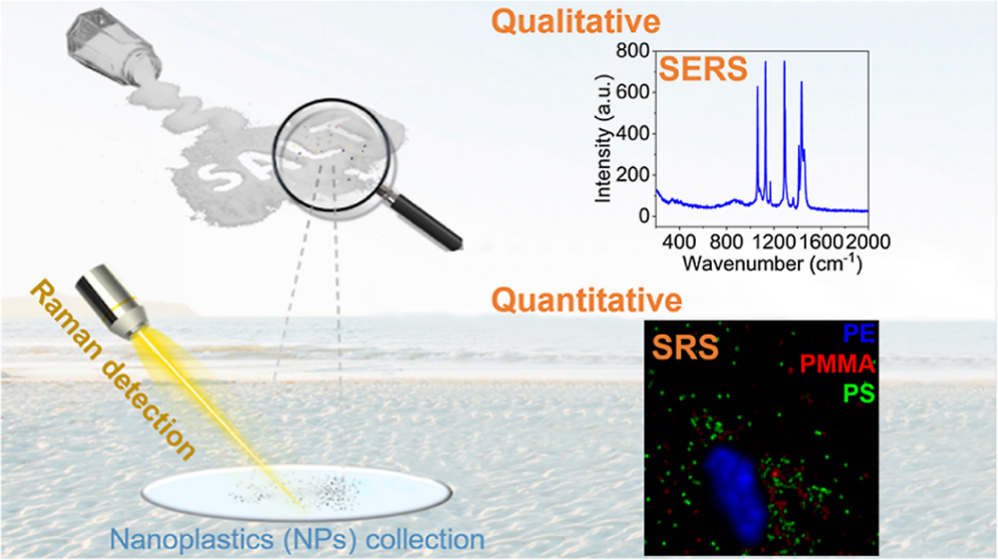

People of all ages consume salt every day, but is it really just salt? Plastic nanoparticles [nanoplastics (NPs)] pose an increasing environmental threat and have begun to contaminate everyday salt in consumer goods. Herein, we developed a combined surface enhanced Raman scattering (SERS) and stimulated Raman scattering (SRS) approach that can realize the filtration, enrichment, and detection of NPs in commercial salt. The Au-loaded (50 nm) anodic alumina oxide substrate was used as the SERS substrate to explore the potential types of NP contaminants in salts. SRS was used to conduct imaging and quantify the presence of the NPs. SRS detection was successfully established through standard plastics, and NPs were identified through the match of the hydrocarbon group of the nanoparticles. Simultaneously, the NPs were quantified based on the high spatial resolution and rapid imaging of the SRS imaging platform. NPs in sea salts produced in Asia, Australasia, Europe, and the Atlantic were studied. We estimate that, depending on the location, an average person could be ingesting as many as 6 million NPs per year through the consumption of sea salt alone. The potential health hazards associated with NP ingestion should not be underestimated.

## Abstract 2

This study demonstrates the first qualitative and quantitative detection of NPs from sea salts by combining SERS and SRS.

## 1 Introduction

Plastic pollution is one of the most acute environmental concerns in the world today.^1−3^ Although the natural degradation of plastic takes a long time, biological,^4^ mechanical wear,^5^ ultraviolet light,^6^ high temperature,^7^ and other factors can cause larger plastics to break down into microplastics (MPs) and nanoplastics (NPs) relatively quickly. Many studies have shown that micro/nanoparticles can enter the lymphatic and circulatory systems of humans (particle size: 0.2–150 μm), rabbits (0.1–10 μm), and dogs (3–100 μm) via living cells.^8−10^ Often, entry is via Peyer’s patch in the intestine.^10,11^ Although 90% of MPs ingested by the human body are excreted in the feces, 10% are absorbed into the bloodstream; because plastics are nearly nondegradable, they have the potential to bioaccumulate in secondary organs and affect the immune system and cell function.^11−18^ NPs can also cross the blood-to-brain barrier and cause behavioral disorders.^19^ In terms of size, NPs are close to natural proteins, and they tend to adsorb organic matter, metals, some nonmetals, and additives/monomers.^20−23^ NPs pose a health risk because they are small enough to easily cross biological membranes through passive diffusion and some endocytosis pathways.^24^

MPs were also detected in food and food utensils. Li et al. investigated the potential exposure of infants to MPs from consuming formula prepared in polypropylene (PP) infant feeding bottles.^25^ Hernandez et al. found that plastic teabags can release billions of MPs, and the authors’ initial invertebrate toxicity assessment showed dose-dependent behavioral and developmental effects.^7^ People consume salt every day in a variety of ways, and there have recently been many reports on MPs in salt^26−28^ using a variety of detection methods, such as Fourier transform infrared,^27^ scanning electron microscopy-X-ray energy dispersive (SEM–EDX),^29^ and Raman spectroscopy.^30^ Being substantially smaller than MPs, NPs can pass through biological membranes and readily translocate between different tissues, leading to significantly more potent toxicological consequences. However, due to detection technology limitations, NPs have rarely been studied in food, and related information is scarce.

Surface-enhanced Raman scattering (SERS) provides a simple and rapid method to study NPs. Due to the enhancement effect (EF) of the electromagnetic field,^31^ a single NP located in a “hotspot” can benefit from a large EF.^30^ So far, SERS has been applied to the study of individual atmospheric aerosol,^31^ NPs,^30^ etc. Our previous study has shown the potential of SERS in detecting NPs in the environment by using a commercial substrate (Klarite).^30^ However, Klarite is not very suitable to study samples of analytes in low concentrations in solution because of the difficulty in transferring samples onto the SERS substrate. Moreover, it still presents a great challenge in the quantitative study of NPs by SERS due to the slowness of Raman imaging. By contrast, stimulated Raman scattering (SRS), as a label-free imaging technique, has been widely employed for rapid imaging of living cells and tissues,^32−36^ as well as for the chemical analysis of individual atmospheric aerosols.^37^ The features of spectral profiles identical to spontaneous Raman spectroscopy, high spatial resolution, and rapid imaging render SRS microscopy a potentially robust method for quantitative study of NPs through chemical imaging.

Herein, by combining SERS (using a developed SERS substrate with a filtering function) and SRS, we demonstrate the first qualitative and quantitative detection of NPs from sea salts. A gold nanoparticle-coated anodic alumina oxide (AAO) membrane is developed as a SERS substrate for NP detection. The SERS substrate is composed of ordered, dense, circular holes with a diameter of 250 nm. This membrane serves to simultaneously filtrate, collect, and detect the NPs. We investigated NPs in sea salt that were collected from different countries. Our samples include 6 source locations covering Asia, Australasia, Europe, and the Atlantic. The NPs in sea salt are further quantified by SRS imaging.

## 2 Experimental Section

### 2.1 Precautions to Prevent Contamination of Samples

To avoid potential NP contamination, all laboratory appliances used are made of clean glass. The researchers wore 100% cotton lab coats with nongranular nitrile gloves; lab coat sleeves were secured inside the gloves. Laboratory equipment was cleaned thoroughly before use. In the qualitative and quantitative analyses of sea salt samples, to avoid errors caused by possible pollution in the overall experimental process, the blank control experiment was completely synchronized with the actual sample experiment, and the experimental results were compared and analyzed.

### 2.2 Polystyrene, poly(methyl methacrylate), polyethylene, PVC, polyethylene terephthalate (PET), and PP

Polystyrene (PS) spheres with diameters of 360 nm, 500 nm, 1 μm, 2 μm, and 5 μm, and poly(methyl methacrylate) (PMMA) spheres with diameters of 360 nm, 500 nm, 2 μm, and 5 μm dispersed in deionized water at 5% (w/v) were purchased from Shanghai Huge Biotech Co, China. The mass density of the PS material is 1.05 g/cm^3^. To obtain individual particles, both PS and PMMA spheres were diluted with deionized water to a ratio of 1:4 × 10^4^ in a volume of 4 mL. The final concentration of plastic particles is 2.625 × 10^–5^ g/cm^–3^. Compared with PS and PMMA, it is more difficult to get model PE, PE terephthalate (PET), polyvinyl chloride (PVC), and PP NPs with a specific size. PE, PET, PVC, and PP NPs models were studied from commercial powder products. PE, PET, and PP are irregularly shaped particles, while PVC has a spherical shape, as shown in Figure S3.

### 2.3 Au-AAO SERS Membrane Fabrication

AAO was purchased from Shenzhen Top Membranes Technology Co., Ltd. Au nanoparticles with different thicknesses on the front of AAO were sputtered with an ion sputtering instrument (Beijing Gewei Technology Co., Ltd., GVC-1000), and the thickness of Au was denoted as *X*. Au-AAO-*X* (*X* = 0, 10, 20, 30, 40, 50, 60, 70, 80, 90, 100 nm). For the performance evaluation of the Au-AAO membrane, the solution containing standard PS and PMMA was dropped on different Au thicknesses of AAO membrane using a glass pipet gun and dried at room temperature. The AAO substrate is placed on the slide and then on the stage of the XploRA Plus confocal Raman spectrometer. Since environmental samples are usually more complex and often emit fluorescence,^38^ the laser wavelength of 785 nm was selected to reduce fluorescence. Laser wavelengths of 532 and 633 nm tend to yield higher fluorescence with these samples.

### 2.4 Treatment and Measurement of Sea Salt Samples

Samples no. 2, 5–6 were purchased from Jing Dong and Tao Bao (online supermarkets), sample no. 1 was purchased from supermarkets in China, and samples no. 3–4 were purchased from the UK. The sources of these 6 kinds of salt are from the Huai salt production areas of China (no. 1), the frigid current between Australia and Antarctica (no. 2), the Mediterranean Sea (no. 3), the coast of France near the North Atlantic Ocean (no. 4), the Seto Inland Sea of Japan (no. 5), and the Sinan Sea of Korea (no. 6).

First, 200 g of sea salt was dissolved in 3000 mL of deionized water. After the sea salt was completely dissolved, a cellulose filter membrane (with a pore size of 1 μm and a diameter of 47 mm) was used for filtration to remove particles with sizes greater than 1 μm in the sea salt. The filtrate with particles smaller than 1000 nm was further filtered on the cellulose filter membrane with a pore size of 0.2 μm. After the filtration, the filter membrane was placed in a clean glass beaker for sonication, and the particles of 200–1000 nm were dispersed in deionized water. The hydrogen peroxide^27,30,39^ solution (30%) was added to the sample to eliminate the interference of the Raman spectrum by organic, biological, and other nonplastic substances and left at room temperature for 24 h with a glass plate covering the beaker to avoid contamination. The sample solution was then filtered again on the Au-AAO-50 filter membrane and dried at room temperature. Qualitative tests were performed directly on the above membrane. In the blank experiment, 3000 mL of deionized water was also taken and treated according to the above steps. For SRS quantitative detection, the solution containing the nanoparticles (obtained after sonication) was dropped onto a clean square glass cover slide of 1.8 cm × 1.8 cm so that the solution spread over the entire glass slide and dried at a temperature of 45 °C in an oven. It was then measured using SRS. Three replicate experiments were performed for each sea salt sample. To assess the recovery rate of NPs, 500 nm PS NPs was used as a representation. In practice, the subsequent processing and detection are the same as the sea salt samples. With the complete procedure of Raman measurements, the recovery rate of NPs was 76.1 ± 6.5%.

For quantitative measurements of NPs by SRS, ten square areas (with an area of 1248 μm^2^ for each) were tested (The locations of the tested areas on the glass cover slide are shown in Figure S15). The calculation of sea salt particles is based on eqs 1 and S1 and S2 are defined as the total area of the glass slide and the total area of 10 test areas tested under SRS, respectively. The size of an area is 1248 μm^2^. N1 and N2 refer to the annual intake of NPs by one person and the number of NPs average of three replicate experiments, respectively. W1 and W2 refer to the amount of salt a person eats in a year (5 g × 365 days) and the amount of salt in each sample (Table S1), respectively. Three replicate experiments were performed for each sea salt sample, and the average value was taken for total annual intake calculation.
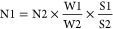
1

### 2.5 Verification of the Quantitative Measurement by SRS

To verify the accuracy of the quantitative method, we dried a known amount of 500 nm PS on a glass slide and tested the amount of PS in 10 test areas using SRS by the same procedure, repeated three times. The results showed that the accuracy could reach more than 80% (Figure S16 and Table S2). To further verify the validity of the method, a 30 areas (Figure S17) measurement were conducted on one of the actual samples (sea salt from Sinan Sea in Korea). The locations of the tested areas on the glass cover slide are shown in Figure S18. The results showed that the number of NPs on the whole glass slide obtained from 30 areas experiment (216,000/200 g) is similar to that of 10 areas experiment (251,000/200 g). It further illustrates the feasibility of our SRS quantitative measurement procedure with 10 testing areas.

### 2.6 Raman Microspectroscopy

Raman spectroscopy was characterized by a XploRA-Plus confocal Raman spectrometer (Jobin Yvon, HORIBA Gr, France) combined with ×100 and ×50 mm Olympus microscope objective (Olympus, 0.90 Numerical Aperture). The external-cavity diode laser (785 nm) with a power of 25 mw was used to excite the sample. The diffraction grating density and blaze wavelengths are 1200 lines per millimeter and 750 nm, respectively. A multichannel EMCCD device with the confocal imaging of 0.5 μm *XY* was used for spectral detection, the resolution of 1.4 cm^–1^ full widths at half maxima. The spectrum collection range was from 200 to 2000 cm^–1^. The acquisition time and spectra accumulations of PS, PMMA standard samples, and food samples were 20 s, 4; 50 s, 4; 50 s, and 4, respectively. Raman mapping was performed using point-by-point scanning with a step size of 300 nm, and the mapping region is 4 μm × 4 μm.

### 2.7 Numerical Simulations

Finite-Difference Time-Domain (FDTD) simulations revealing the electric field distribution on the surface of the Au-AAO-50 SERS substrate were performed in Lumerical. Guided by SEM and atomic force microscopy (AFM) of the surface, the model SERS substrate was created in Autodesk Inventor and imported into Lumerical. The model was generated from a bicentric hexagonal pattern of holes with side lengths of 500 nm and hole diameters of 250 nm. The orifices were tapered to the surface and resembled an array of countersunk holes with a chamfer angle of 84° and a diameter of 345 nm at the surface. The AAO was modeled using a wavelength dependent material model in the Lumerical database from refs (40 and 41). A 50 nm layer of Au was superimposed onto the surface of the AAO substrate and modeled using a Johnson and Christy material model (based on ref (42)) to simulate the substrates used in experiments reported here. Three other metallic layers were modeled for comparison, Ag (Palik 0–2 μm material model; ref (43)), Al, and Cu (both CRC material models; ref (44)).

The simulation domain space encompassed 2 μm × 1.732 μm of the surface (*x* and *y* directions) of the substrate and a depth of 2 μm (*z* direction). 3 μm void space was placed above the substrate surface. The boundaries of the domain were periodic in the *x* and *y* directions and perfectly matched layers in the *z* direction. The mesh resolution was approximately 2% of the hole diameter.

A Bloch plane wave source with an amplitude of 1 V/m was introduced from 2 μm above the SERS substrate model. The source was a Fourier transform limited pulse of light with an approximately Gaussian spectral profile centered at 785 nm with a wavelength span of 300 nm. The plane wave was linearly polarized parallel to the *x*-axis of the FDTD domain. A frequency domain field and power monitor were placed in the *x*–*y* plane 1 nm above the surface of the SERS substrate to extract the electric field distribution on the surface of the sample.

### 2.8 Enhancement Factor

We quantified the SERS EFs according to the following formula
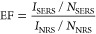
2*I*_SERS_ and *I*_NRS_ are defined as the peak intensities detected by the SERS substrate (Au-AAO-50) and non-SERS AAO substrate, respectively; *N*_SERS_ and *N*_NRS_ refer to the number of molecules contributing to SERS and non-SERS Raman peak intensities, respectively. In this study, single isolated particles were measured, so *N*_SERS_ and *N*_NRS_ are fixed at *N* = 1. To eliminate the effects of accumulation time and laser power on the measured Raman strength, these parameters are kept constant. After removing the baseline spectrum of the substrate, the peak height of Raman intensity was measured.^30^ Five spheres of each size were randomly selected for measurement to avoid the influence of fluctuation and ensure signal stability for further study. The peak at 1003 cm^–1^ (PS) and 812 cm^–1^ (PMMA) were used to calculate the EF.

### 2.9 SRS Microscopy

A commercial optical parametric oscillator (OPO) laser system (Insight DS+, Spectra-Physics, Newport) was employed to provide two pulsed femtosecond lasers with an 80 MHz repetition rate. The fundamental 1040 nm laser (30 mW) was severed as a Stokes beam, and the OPO output was set at 804 nm (15 mW), regarded as a pump beam. To reach better spectral resolution (13 cm^–1^), the pulse width of both beams was chirped and stretched to ∼1.8 ps with SF57 glass rods. The intensity of the Stokes beam was modulated by an electro-optical modulator (EO-AM-R-20-C2, Thorlabs) at 20 MHz with >90% modulation depth. After being spatially overlapped through a dichroic mirror, both lasers were guided into a laser scanning microscope (FV1200, OLYMPUS) and focused onto the sample by a 60× water immersion objective lens (N.A. 1.2, Olympus). Passing through the sample, the transmission beams were collected by a high N.A. oil condenser (N.A. 1.4, Nikon), optically filtered (CARS ET890/220, Chroma), and detected by a homemade back-biased photodiode. At last, the stimulated Raman loss signal was demodulated by a lock-in amplifier (HF2LI, Zurich Instruments) and fed to form SRS images. In our spectral-focusing mode, a motorized stage (M-ILS250CC, Newport) was applied to tune the time delay between pump and Stokes beams for SRS spectral acquisition.

### 2.10 Data Processing

LabSpec 6, Origin 2016, PowerPoint 2021, Photoshop 2019, and ImageJ 2015 software were used to process the data and generate figures.

## 3 Results and Discussion

### 3.1 Characterization of Au-AAO-50

**Figure 1:**
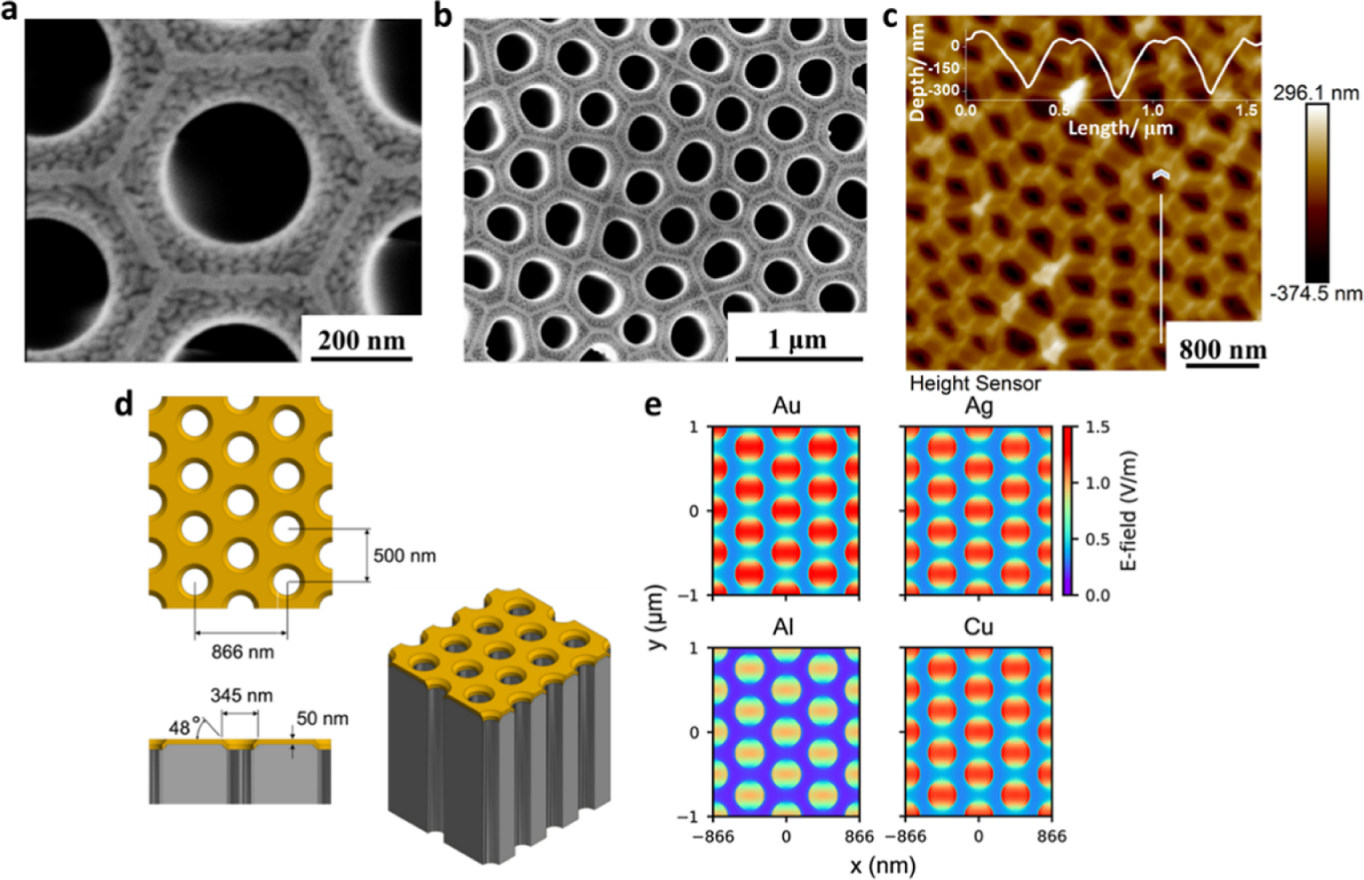
Structure and computational simulation of Au-AAO-50 membranes. (a,b), SEM images of Au-AAO-50. (c) AFM images of Au-AAO-50. (d) Simulation model of Au-AAO-50. (e) Comparison of the electric near-fields on the substrate surface depending on the metallic material used to coat the AAO substrate obtained from FDTD simulations. The four panels present the amplitude of the electric field in the *x*–*y* plane for Au (top left), Ag (top right), Al (bottom left), and Cu (bottom right) simulated with a normal incident pulse of light centered at 785 nm.

The SERS substrate for filtrating and detecting NPs was first geometrically characterized, and then, the electric field distribution on the substrate was simulated. The SERS substrate was fabricated by ion sputtering Au nanoparticles on the upper surface of the AAO membrane. The thickness of sputtered Au was optimized at 50 nm (discussed later); the corresponding sample is referred to as Au-AAO-50. This sample (Au-AAO-50) was studied by SEM and AFM, as shown in Figure 1.

The pores are about 250 nm in diameter on average. It can be observed by SEM that the size of gold nanoparticles attached to the upper surface of AAO is about 20 nm-50 nm. The structure of the Au layer on the AAO substrate, especially its texture near the pores, was investigated by AFM. A funneled porous sieve shaped Au film surrounding the pores was observed (Figures 1a and S1). The optical properties and electric field distributions of the Au-AAO-50 membrane were further simulated with a Maxwell’s equations solver (Lumerical), based on the geometrical measurements obtained from SEM and AFM (Figure 1d,e). Figure 1e presents the simulated electric near-field distribution on the surface of the AAO-SERS substrate for four different metallic nanolayers: Au, Ag, Al, and Cu. In each case, the electric field strength is greatest at the center of the holes, where the plastic particles are most likely to reside after filtration. As a result of the light polarization (oriented along the *x*-direction), the electric field within the holes appears to be greatest along the *x*-axis, across the center of the holes; the electric field strength at the upper and lower edges of the holes is weaker. Above the surface of the metallic layer surrounding the holes, the electric field strength is close to zero. Au showed the greatest enhancement of the electric field within the holes at 785 nm. Ag and Cu were almost comparable. The Al layer yielded the weakest result at 785 nm by far. The simulation results suggest that Au would be best suited for generating enhanced electric fields at the center of the holes, to benefit SERS.

### 3.2 Performance of Au-AAO-50

**Figure 2:**
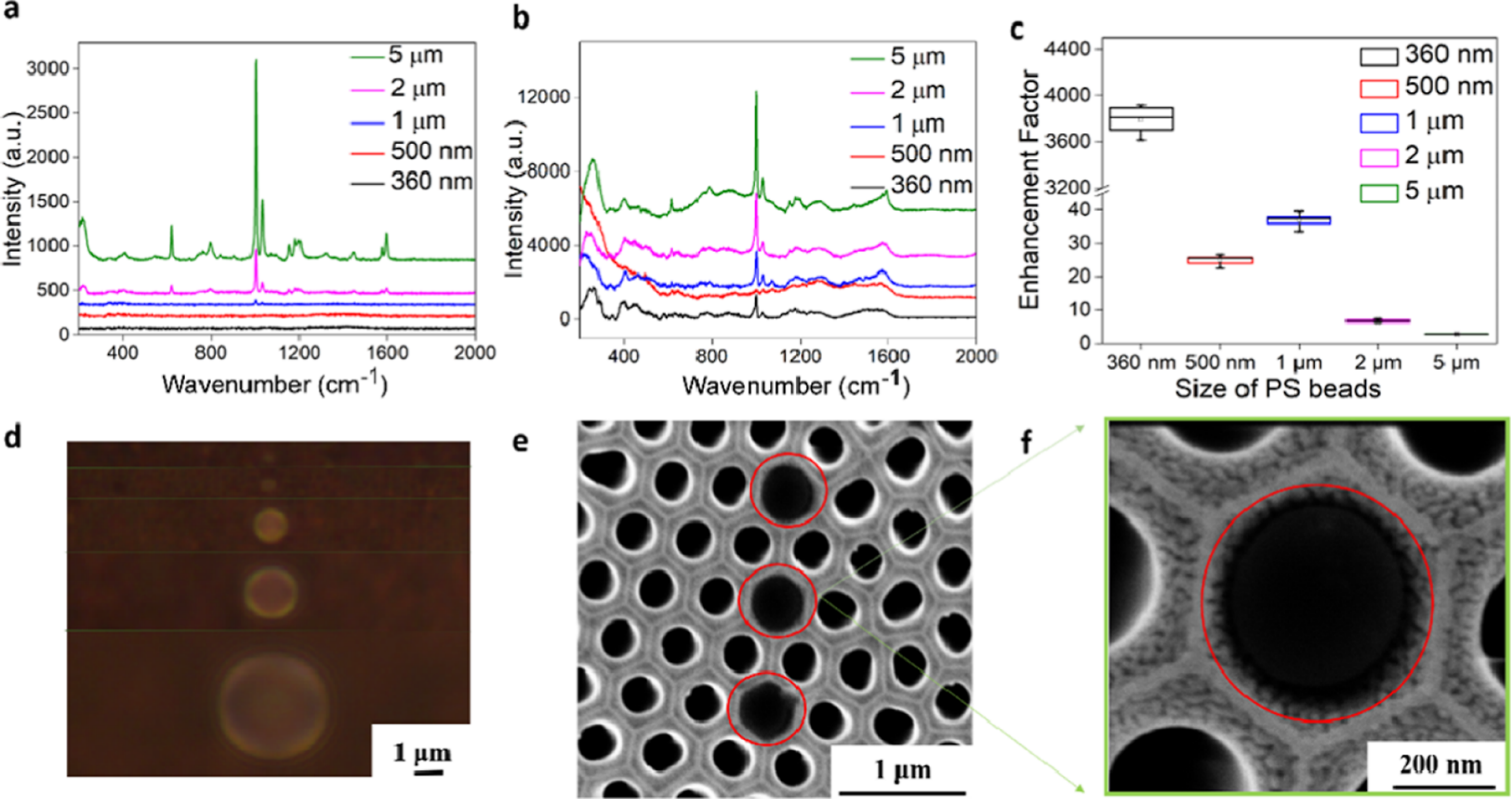
Raman signals, EFs, and imaging of PS spheres on membranes. (a,b) Raman spectra of PS spheres of a variable size on AAO (a) and Au-AAO-50 (b) (4 × 20 s spectral acquisitions). (c) Box and whisker plot of EFs of PS particles as a function of size. (d) Bright field microscopy images of PS spheres with sizes of 360 nm, 500 nm, 1 μm, 2, and 5 μm placed on the Au-AAO-50 substrate. (e,f) SEM images of 360 nm PS spheres on Au-AAO-50.

The performance of the SERS substrate is evaluated first by detecting PS, and Raman signals of 5, 2, and, 1 μm PS spheres can be reliably detected on a non-SERS AAO substrate; the signal of PS spheres smaller than 1 μm is barely observable. However, on Au-AAO-50, the Raman signal of a single PS sphere of all sizes (minimum studied diameter 360 nm) can be detected (Figure 2b). The two most significant peaks (at 1003 and 1033 cm^–1^) are attributed to the ring-mode vibrations of the monosubstituted aromatic compound [v(C–C) and β(C–H)] in PS.^30^ Therefore, in sharp contrast to the sample on AAO, PS spheres smaller than 1 μm can be identified in Raman spectra on the Au-AAO-50 substrate. This result demonstrates the strong potential of Au-AAO-50 to enhance the intensity of Raman signals in the weak Raman scattering samples. On the Au-AAO-50 substrate, the 500 nm PS spheres also show significantly enhanced Raman peaks, although the peak intensity is not as high as that of the 360 nm PS spheres, indicating efficient enhancement for smaller NPs. The optimum thickness of the Au film on the AAO is also studied. Figure S2 shows the Raman spectra of PS spheres on Au-AAO with Au thickness from 10 to 100 nm. With the increase of the sputtering amount of Au from 10 to 50 nm, more Au particles exist on the substrate surface, which reduce the Au particle gap and can provide more “hot spots” that can enhance the local electric field intensity. Therefore, the Raman signal is significantly enhanced. However, when the thickness of sputtered Au is >50 nm, the gap of Au particles gradually decreases and even disappears. Accordingly, the number of “hot spots” decreases, which reduces the SERS effect. The best performance is observed when the thickness of Au film is around 50 nm; therefore, Au-AAO-50 is used throughout this study.

Figure 2c shows the calculated EFs of the SERS substrate for PS detection. The EF for 360 nm PS spheres is an order of magnitude larger than our previous work using a commercial SERS substrate (Klarite),^30^ indicating the superior performance of the Au-AAO-50 substrate for NP detection. The average EF for PS with a diameter of 360 nm is 3785.45 ± 113.01. The average EF of 500 nm PS spheres is 24.80 ± 1.42, which is significantly smaller than that of 360 nm spheres. The EF of 1 μm PS spheres is slightly higher than that of 500 nm PS spheres, with an average value of 36.81 ± 2.03. Figure 2d shows an image of several PS spheres with different sizes (ranging from 360 nm to 5 μm) under an optical microscope that is integrated within our Raman instrument. Figure 2e,f are SEM images of 360 nm PS spheres. The images clearly show that these 360 nm PS spheres lodged exactly above the holes of the SERS substrate. As shown in the electric field distribution simulation (Figure 1e), the electric field within the holes appears to be greatest, which points to the likely origin of the Raman signal enhancement.

**Figure 3:**
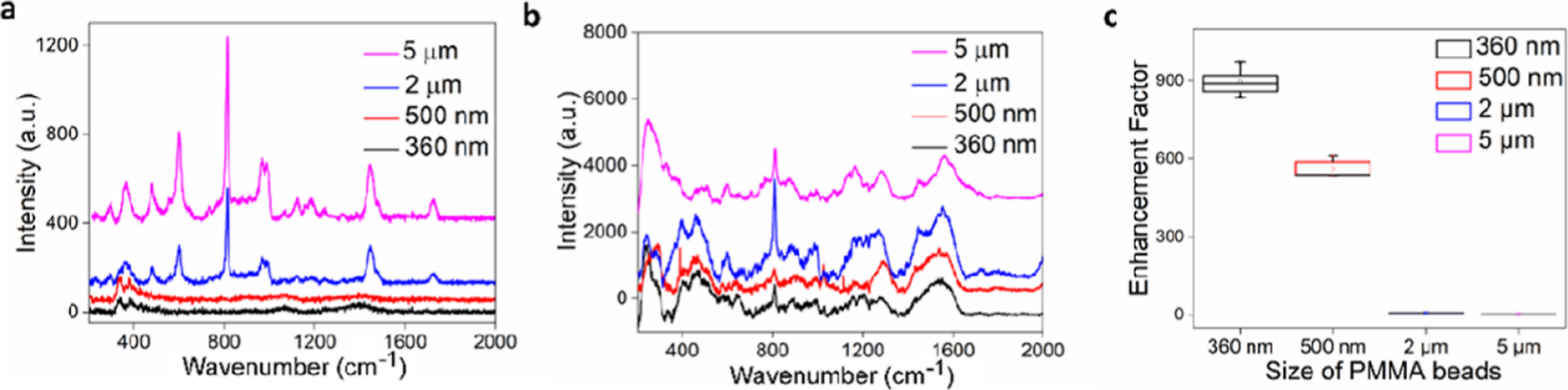
Raman signals and EFs of PMMA. (a,b) Raman spectra of PMMA spheres with different particle sizes on AAO (a) and Au-AAO-50 (b) (4 × 50 s spectral acquisitions). (c) Box and whisker plot of EFs of PMMA spheres as a function of size.

To further verify the versatility of the Au-AAO-50 substrate for the SERS detection of M/NPs, PMMA NPs were also studied. PMMA is widely used to replace glass, instrument parts, car lights, optical lenses, transparent pipes, bathtubs, washbasins, and other products. The Raman spectra of PMMA spheres on the non-SERS AAO substrate are shown in Figure 3a. For PMMA spheres with sizes smaller than 2 μm, hardly any PMMA Raman signature can be detected. However, for PMMA spheres on the Au-AAO-50 SERS substrate (Figure 3b), PMMA spheres as small as 500 and 360 nm can be readily detected. The peaks at 600, 812, 988,1445, and 1726 cm^–1^ were visible and attributed to C–C–O stretching, C–O–C symmetric stretching, O–CH_3_ rocking, C–H bending, and C=O stretching, respectively. The Raman peak at 812 cm^–1^ was the strongest, so the peak at 812 cm^–1^ was selected to calculate the EF. Figure 3c shows a box and whisker plot of the EF as a function of particle size. The SERS substrate presents the strongest EF for PMMA spheres of 360 nm, ranging from 836.89 to 971.60. For 500 nm PMMA spheres, the EF is slightly lower, ranging from 534.39 to 612.21. The EF increases when the size of the PMMA sphere decreases, showing a similar trend to that of the PS sphere. For both PS and PMMA particles, the EFs are an order of magnitude larger compared to previous work.^31^ In addition, due to its porous structure, the substrate has a filtering function that enriches it in nanoparticles, which Klarite lacks. Nanosized PE, PVC, PET, and PP were able to be detected by our SERS method, and the results are also compared with the corresponding microsized particles, as shown in Figure S3.

### 3.3 Qualitative Study of NPs in Sea Salt

**Figure 4:**
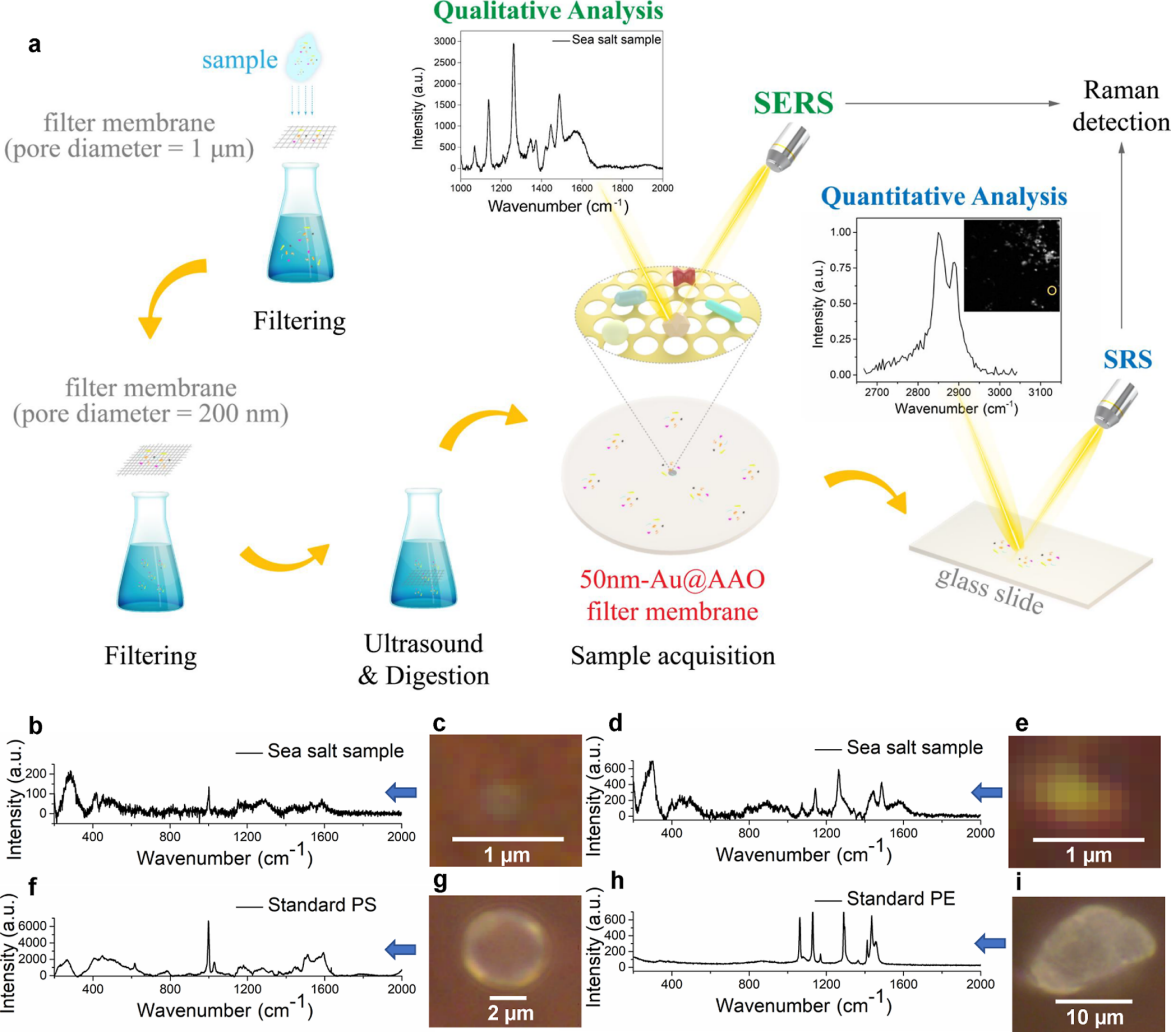
Qualitative detection of NPs in sea salt. A Flowchart of sea salt sample processing. (b,d) Raman spectra of particles identified as PS and PE, respectively, in the sea salt samples (4 × 50 s spectral acquisitions). (f,h) corresponding standard reference spectra of PS and PE, respectively. (c,e) bright field microscopy images of PS and PE particles in sea salt, respectively. (g,i) bright field microscopy images of PS and PE standard samples, respectively.

NPs were extracted from the sea salt samples and detected using Au-AAO-50 SERS substrates. First, solid sea salt was dissolved in deionized water. The solution was then filtered by a filter membrane with a pore size of 1 μm to remove the larger particles. The filtrate was further filtered by the porous Au-AAO-50 SERS membrane (Figure 4a). Therefore, nanoparticles including NPs with sizes in the range 0.2–1 μm were collected and then tested. Figure 4b,d shows the Raman spectra of typical particles extracted from sea salt on Au-AAO-50. We found that the spectra of some NPs in sea salt matched well with PE, others with PS; the particles tested are shown in Figure 4c,e. The Raman spectrum of PE has vibrational modes at 1070, 1134, 1280, 1374, and 1446 cm^–1^, that are attributed to C–C stretching, C–C stretching, CH_2_ twisting, CH_2_ wagging, and CH_2_ bending, respectively. A PS particle was also detected by Raman mapping (Figure S4).

### 3.4 Quantitative Study of NPs in Sea Salt

Our next step was to make a quantitative study of these plastic NPs in sea salt. However, performing such a study using SERS faces a significant challenge–Raman imaging is quite slow. For example, NPs measurements in an area of 16 μm^2^ on the SERS membrane took approximately 10 h, as shown in Figure S4a. To speed up the analysis, SRS imaging represents a good alternative. SRS imaging was then used for quantitative analysis of NPs in sea salt, since the type of NPs had already been identified using SERS. With SRS, an area of 1248 μm^2^ took only about 2 min to measure. Herein, SERS was first used to identify the types of NP contaminants in sea salt samples, while SRS was used for quantification and imaging of the NPs. Specifically, SERS was used to obtain the characteristic Raman spectra of the NPs, which can serve as a reference for subsequent SRS imaging and quantification. By using SERS, we are able to identify the presence of NPs such as PS and PE in the sea salt samples. Subsequently, SRS was used to conduct imaging and quantify the presence of these NPs in the samples, which provided more detailed and quantitative information about the NPs.

**Figure 5:**
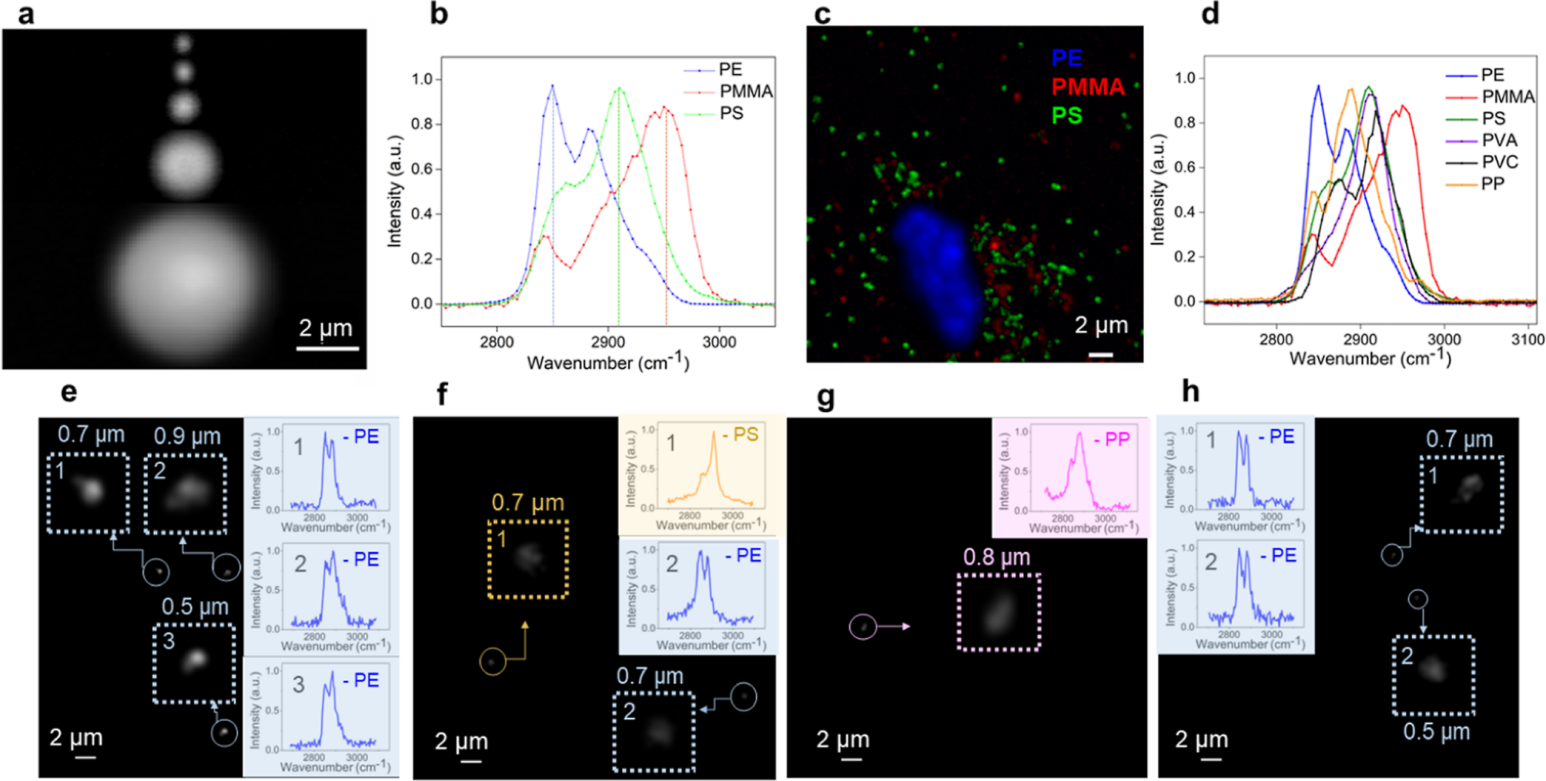
Plastic particles measured with SRS microscopy. (a) SRS images of PS particles with sizes of 5 μm, 2 μm, 1 μm, 500 nm, and 360 nm, respectively. (b) SRS spectra of characteristic peaks of PS, PE, and PMMA. (c) SRS image of mixed PE, PMMA, and PS samples. (d) SRS spectra of standard plastic samples. (e–h) SRS imaging and spectra of NPs in sea salt samples.

Single PS spheres with sizes ranging from 360 nm to 5 μm can be successfully detected with SRS imaging, as shown in Figure 5a. The characteristic peaks of PS at 2910 cm^–1^, PE at 2850 cm^–1^, and PMMA at 2950 cm^–1^ were selected as the discriminative peaks for SRS imaging (Figure 5b). Figure 5c presents an SRS image of a sample containing a mixture of PE, PMMA, and PS. Clearly, different types of NPs can be accurately discriminated by SRS imaging. Plastics in sea salt samples are not well-known and can be diverse. For plastics with overlapping peaks at high wavenumber scanning, the sample will be further checked based on the peak shape or low wavenumber measurement. The detection efficiency would be slowed down by continuously adjusting pump wavelength to match SRS resonance conditions at multicharacteristic sharp peaks of different plastics. Therefore, we proposed a detection method to improve efficiency based on the results that SRS image at 2865 cm^–1^. The method can reveal numerous kinds of plastics, as the hydrocarbon group is a fundamental component for most plastics. We first screened the samples with pump beam tuned at 804 nm, covering the broad band of 2800–3000 cm^–1^ along with 1040 nm Stokes beam. Then hyperspectral scanning was performed to identify different types of plastics based on their characteristic spectral lineshapes. Standard samples of different plastics were measured with SRS to obtain the SRS reference signals, and the signals of the actual particle measured in sea salt were compared with those of the reference. Evaluations of SRS measurement of model PS, PMMA, PE, PVC, poly(vinyl alcohol) (PVA), and PP were also performed (Figure 5a–d). The following SRS peaks were used to determine the NPs: PS (2866, 2910 cm^–1^), PE (2850, 2882 cm^–1^), PVA (2910 cm^–1^), PVC (2874, 2918 cm^–1^), PMMA (2842, 2950 cm^–1^), and PP (2842, 2886 cm^–1^), as shown in Figure 5d. Typical examples of SRS imaging and spectra of NPs in sea salt samples are shown in Figure 5e–h. Blank experiments were also performed using an identical method, and no particles with plastic Raman signals were detected. We also studied typical examples of potential NPs in sea salt by SEM (Figure S5), showing particles with size smaller than 1 μm composed of C and O elements, consistent with Raman results.

Notably, for the NPs having the similar SRS spectra, additional spectral unmixing analysis were performed by Gaussian fitting. For instance, we performed a spectral decomposition analysis of the SRS spectra of particle 1 in Figure S9c (Figure S6a), where the peak splitting results are at 2853 and 2910 cm^–1^. This spectrum matches well with that of standard PS in the high band (Figure S7a), with an *R*^2^ value of 0.987. When we assigned it to PVA (Figure S6b), we obtained an *R*^2^ value of 0.878, leading to the identification of particle 1 in Figure S9c as PS instead of PVA. Additionally, for the last particle in Figure S10b, the spectral decomposition analysis of the SRS spectra assigned it to PE with an *R*^2^ value of 0.984 (Figure S6c). If we had assigned it to PP, the third peak position would have been at 2889 cm^–1^, which is misaligned by 67 cm^–1^ with the position for standard PP at 2956 cm^–1^ (Figure S7c), suggesting that this particle is much closer to PE than PP. Similarly, for particles 2 and 3 in Figure 5e, their spectra exhibit a notable concordance with the standard PE spectrum, as illustrated in Figure S7b. The *R*^2^ values for these associations are 0.964 and 0.961, respectively (Figure S6g,h), attesting to a high degree of correlation. In contrast, when particles 2 and 3 were matched with the standard PP spectrum (Figure S7c), the third peak position would be deviated by 69 and 73 cm^–1^ from the standard PP position at 2956 cm^–1^, respectively (Figure S7c). This misalignment suggests a closer affinity of these particles to PE rather than PP. Furthermore, we performed similar procedures for the spectral decomposition analysis of the SRS spectra of the first particle in Figure S10b and the sixth particle in Figure S10c, which were both identified as PP (Figure S6e,f).

NPs in sea salts produced from different locations were then studied; sea salts were collected from supermarkets spanning 6 regions including Asia, Australasia, Europe and the Atlantic. Detailed information on the sources of the sea salts are shown in Table S1. The number of NPs detected in the sea salts from the Mediterranean Sea (no. 3, detailed in Figure S8), the Huai salt production areas of China (no. 1, Figure S9), the Sinan Sea of Korea (no. 6, Figure S10), the frigid current between Australia and Antarctica (no. 2, Figure S11), the Seto Inland Sea in Japan (no. 5, Figure S12), and the coast of France near the North Atlantic Ocean (no. 4, Figure S13) are shown in Table S1 and Figure S14. Based on our estimate of an adult intake of 5 g of salt per day (the amount recommended by the World Health Organization), the amounts of NPs that people in different regions might ingest through sea salt per year were calculated and illustrated in Figure S19. PE and PP are the most frequently detected NPs in sea salt, potentially because they are the most demanded and most used polymer types,^45,46^ and they are the most common polymers found in seawater.^47−49^

If we assume that all daily salt intake of adults is from sea salt (upper limit), in some regions, a person could be consuming up to 6 million NPs (200 nm–1 μm) per year. It is worth noting that NPs with sizes smaller than 200 nm are not counted due to the current limitations of our method. According to the topographic analyses, sea salts with high NPs are mostly produced in bays close to human activity, such as near the Mediterranean, the Huai salt region of China, and the Sinan Sea in Korea. Probably, the water in these regions is highly influenced by human activities that cause significant plastic pollution and end up contaminating sea salt. The poor mobility of the seawater in the bay is a likely aggravating factor.

## 4 Environmental Implication

The universality of NPs in food and food utensils highlights the urgent need for effective detection strategies.^25,50−53^ However, the current detection of plastic particles mainly focuses on the micrometer size range owing to the optical diffraction limit.^26−28,54,55^ Many studies have recently been reported on MPs detected in salt, a necessity for human life,^26−28^ and further research on NPs is necessary to fully understand food plastic pollution. Weak signal and low SNR are major challenges in Raman detection of NPs, and the SERS commercial substrate (Klarite) has the potential for NP single particle detection due to the EF of electromagnetic fields.^30,31^ However, Klarite is not suitable for samples with low concentrations of particles due to difficulties in sample transfer. In addition, limited by imaging speed, quantitative SERS research on NPs still faces huge challenges. Therefore, further research is needed to optimize NP Raman detection substrates and develop quantitative techniques to identify NPs in food samples.

In summary, our work developed and optimized a SERS substrate (Au-AAO-50) that can enrich nanoparticles in salt and achieved qualitative and quantitative measurement of NPs through the combination of SERS and SRS, providing a novel method for the quantitative detection of NPs. During the process of sample detection, the detection speed of SRS is much faster than that of SERS. Therefore, the combination of SERS and SRS overcomes the slow-speed limitation of traditional quantitative methods for NPs. In our study, the Au-AAO-50 film greatly enhanced the Raman signal; the average EF for PS with a diameter of 360 nm is 3785.45 ± 113.01. Therefore, individual nanoparticles were sensitively detected on the Au-AAO-50 substrate. We estimate that, for a person who consumes 5 g of sea salt a day, up to about 6 million NPs would be ingested per year.

As a novel contribution, our work provides an important reference for the qualitative and rapid quantitative detection of NPs in low-concentration food samples. We performed SERS analysis using Au-AAO-50 to identify potential types of NP contaminants present in commercial salt samples. The SERS analysis provided valuable insight into the types of NPs that may be present in the samples, allowing for a more targeted and efficient SRS analysis. The combination of SERS and SRS allowed for both qualitative and quantitative information to be obtained, resulting in a more comprehensive understanding of NP contamination in the commercial salt samples. Considering the health hazards associated with ingesting NPs, more attention should be devoted to studying the health impact of plastic NPs ingested through sea salt.

## Supporting Information Available

Additional details of the funneled porous sieve-shaped Au film, Raman spectra data of PS spheres on Au-AAO, Raman imaging and spectra of PE, PET, PP, and PVC micro- and nanoparticles, quantitative detection of NPs in sea salt by SERS, SEM of potential NPs in sea salt samples, spectral decomposition analysis of the SRS spectra, SRS imaging and spectra of NPs detected in sea salt samples, and test areas, imaging, and quantification results for SRS study (PDF)
