# Supplementary material for: Nanoplastics Detected in Commercial Sea Salt
Source: Environ Sci Technol. 2024 May 6;58(21):9091–101. doi: 10.1021/acs.est.3c11021 (PMC11196019; doi:10.1021/acs.est.3c11021)
Supplement: Supplementary file 1 — es3c11021_si_001.pdf [file es3c11021_si_001.pdf]

Supplemental information

Nanoplastics Detected in Commercial Sea Salt

Xuejun Ruan[1#], Jianpeng Ao[2#], Minglu Ma[1#], Robin R. Jones[3], Juan Liu[1], Kejian Li[1], Qiuyue Ge[1], Guanjun Xu[1], Yangyang Liu[1], Tao Wang[1], Lifang Xie[1], Wei Wang[1], Wenbo You[1], Licheng Wang[1,] Ventsislav K. Valev[3], Minbiao Ji[2]*, Liwu Zhang[1,4]*

[1]Shanghai Key Laboratory of Atmospheric Particle Pollution and Prevention, National Observations and Research Station for Wetland Ecosystems of the Yangtze Estuary, IRDR international Center of Excellence on Risk Interconnectivity and Governance on Weather, Department of Environmental Science & Engineering, Fudan University, Shanghai, 200433, Peoples' Republic of China.

[2]State Key Laboratory of Surface Physics and Department of Physics, Human Phenome Institute, Academy for Engineering and Technology, Key Laboratory of Micro and Nano Photonic Structures (Ministry of Education), Yiwu Research Institute of Fudan University, Fudan University, Shanghai, 200433, Peoples' Republic of China

[3]Centre for Photonics and Photonic Materials and Centre for Nanoscience and Nanotechnology, Department of Physics, University of Bath, Claverton Down, Bath BA2 7AY, United Kingdom

[4]Shanghai Institute of Pollution Control and Ecological Security, Shanghai, 200092, Peoples' Republic of China.

* Corresponding Authors: Dr. Liwu Zhang (Email: zhanglw@fudan.edu.cn); Dr. Minbiao Ji (Email: minbiaoj@fudan.edu.cn)

Catalog

Fig. S1 (a) Photograph of Au-AAO-50 membrane. (b) Cross-sectional SEM image of the Au-AAO-50 membrane. (c) Schematic drawing of a 360 nm PS on the Au-AAO-50. ....S5

Fig. S2 (a-j) Raman signals of PS spheres of different sizes (360 nm, 500 nm, 1 μm, 2 μm, 5 μm) on Au-AAO membrane with varying thickness of gold. Au-AAO-10 (a), Au-AAO-20 (b), Au-AAO-30 (c), Au-AAO-40 (d), Au-AAO-50 (e), Au-AAO-60 (f), Au-AAO-70 (g), Au-AAO-80 (h), Au-AAO-90 (i) and Au-AAO-100 (j) represent Au-AAO membrane with Au thickness of 10 nm, 20 nm, 30 nm, 40 nm, 50 nm, 60 nm, 70 nm, 80 nm, 90 nm and 100 nm, respectively. S6

Fig. S3 Raman spectra of model nano PE(a), micro PE(e), nano PVC(c), micro PVC(g), nano PET(i), micro PET(m), nano PP (k) and micro PP(o) particles. Microscopy images of model nano PE (b), micro PE (f) nano PVC(d), micro PVC(h), nano PET(j), micro PET(n), nano PP (l) and micro PP(p) particles in bright field. S7

Fig. S4 Quantitative detection of NPs in sea salt by SERS. (a) Raman mapping image of PS in sea salt. (b) Raman spectra of particle identified as PS in the sea salt sample. (c) The reference standard spectra of PS...S8

Fig. S5 Nanoparticles containing C, O elements photographed under SEM. The scale bar is 1 μm. S9

Fig. S6 Spectral decomposition analysis of the SRS spectra. Particle 1 in Fig. S10c identified as PS(a) and PVA(b). The last particle in Fig. S11b identified as PE(c) and PP(d). The first particle in Fig. S11b identified as PP(e). The last particle in Fig. S11c identified as PP(f). Particle 2 and 3 in Fig. 5e identified as PE(g-h). S10

Fig. S7 Spectral decomposition analysis of the SRS spectra of standard PS(a), PE(b), PP(c), PMMA(d), PVA(e), PVC(f). S11

Fig. S8 SRS imaging and spectra of nano PE detected in sea salt samples from No.3 in the Mediterranean region. a, b, and c are the results obtained from three replicate experiments, respectively. S12

Fig. S9 SRS imaging and spectra of nano PE and PS detected in sea salt samples from No.1 Huai Salt production area in China. a, b, and c are the results obtained from three replicate experiments, respectively. S13

Fig. S10 SRS imaging and spectra of nano PE and PP detected in sea salt samples from No.6 Sinan Sea in Korea. a, b, and c are the results obtained from three replicate experiments, respectively. S14

Fig. S11 SRS imaging and spectra of nano PE detected in sea salt samples from No.2 harvested from the frigid current between Australia and Antarctica. a, b, and c are the results obtained from three replicate experiments, respectively. S15

Fig. S12 SRS imaging and spectra of nano PE and PP detected in sea salt samples from No.5 Seto Inland Sea in Japan. a, b, and c are the results obtained from three replicate experiments, respectively. S16

Fig. S13 SRS imaging and spectra of nano PE detected in sea salt samples from No.4 in the French North Atlantic Ocean. a, b, and c are the results obtained from three replicate experiments, respectively. S17

Fig. S14 The amount of nanoplastics contained in 200 g of sea salt. S18

Fig. S15 The locations of 10 test areas on the substrate for SRS study. The size of each square test area is 35.33 μm × 35.33 μm. S19

Fig. S16 Imaging of 10 areas of the SRS swept standard sample, a-c are the results of 3 replicates, respectively. The scale bar is 2 μm. S20

Fig. S17 SRS imaging and spectra of nano PE detected in sea salt samples from No.6 Sinan Sea in Korea. a, b, and c are the results obtained from the experiment was tested 30 areas on one sample, respectively. S21

Fig. S18 The locations of 30 test areas on the substrate for SRS study. The size of each square test area is 35.33 μm × 35.33 μm. S22

Fig. S19 The amounts of NPs that people in different regions would ingest through sea salt per year. Approximate locations of the sources of the sea salts were marked according to brand labels and other available information. S23

Table S1. Summary of actual sample data of sea salt...S24

Table S2. SRS quantification results of a known amount of 500 nm PS to verify the accuracy of 10 testing areas method (repeated three times)..S25

SI References S26

Fig. S1 (a) Photograph of Au-AAO-50 membrane. (b) Cross-sectional SEM image of the Au-AAO-50 membrane. (c) Schematic drawing of a 360 nm PS on the Au-AAO-50.

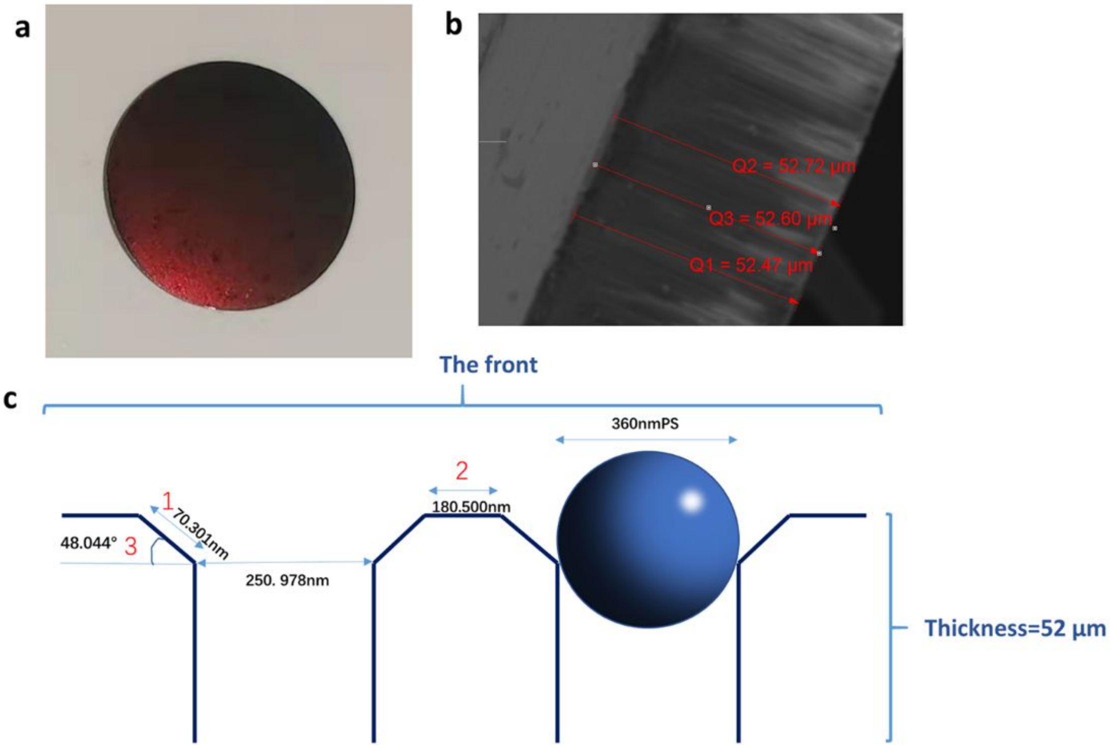

Fig. S2 (a-j) Raman signals of PS spheres of different sizes (360 nm, 500 nm, 1 μm, 2 μm, 5 μm) on Au-AAO membrane with varying thickness of gold. Au-AAO-10 (a), Au-AAO-20 (b), Au-AAO-30 (c), Au-AAO-40 (d), Au-AAO-50 (e), Au-AAO-60 (f), Au-AAO-70 (g), Au-AAO-80 (h), Au-AAO-90 (i) and Au-AAO-100 (j) represent Au-AAO membrane with Au thickness of 10 nm, 20 nm, 30 nm, 40 nm, 50 nm, 60 nm, 70 nm, 80 nm, 90 nm and 100 nm, respectively.

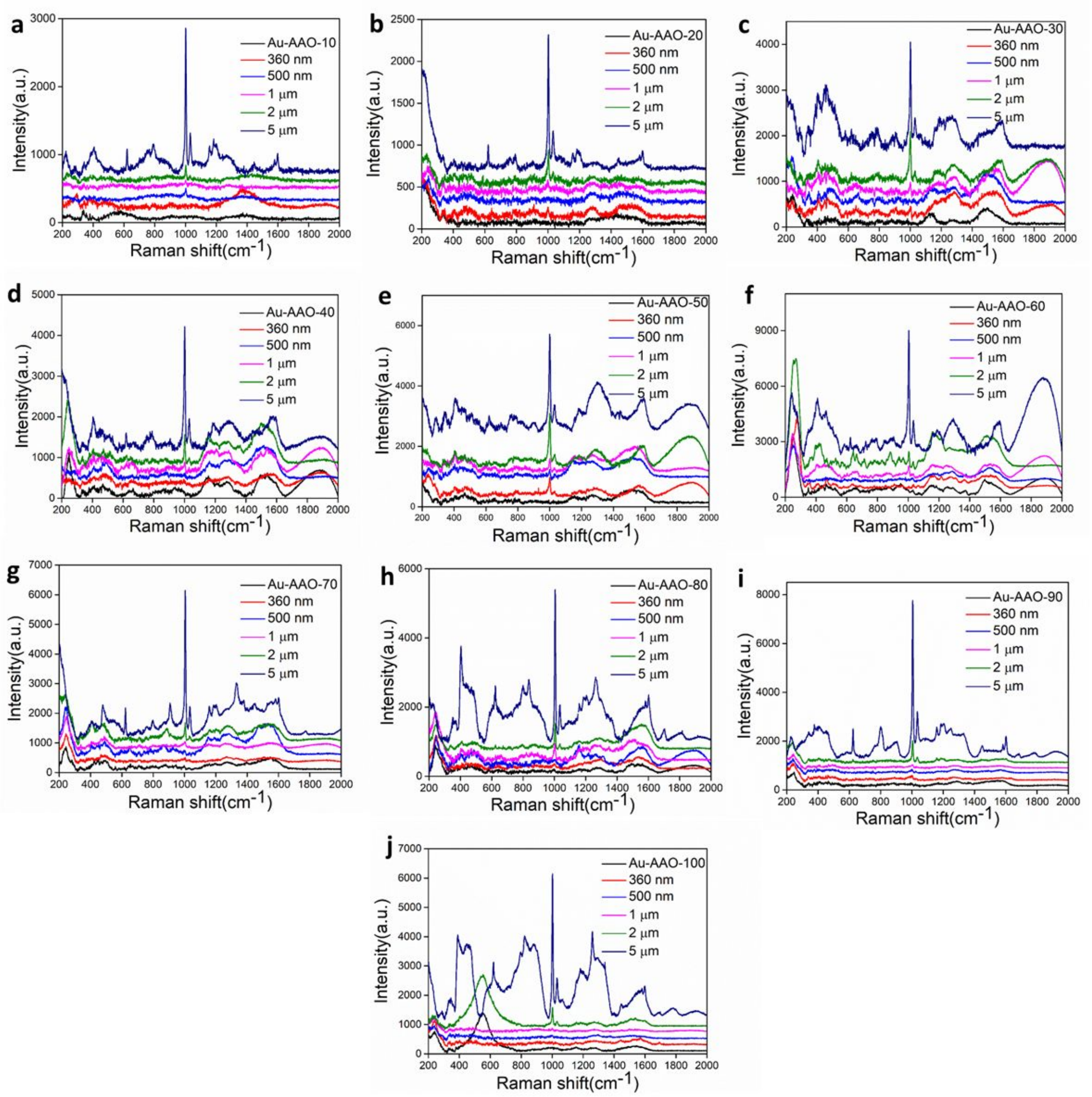

Fig. S3 Raman spectra of model nano PE(a), micro PE(e), nano PVC(c), micro PVC(g), nano PET(i), micro PET(m), nano PP (k) and micro PP(o) particles. Microscopy images of model nano PE (b), micro PE(f) nano PVC(d), micro PVC(h), nano PET(j), micro PET(n), nano PP (l) and micro PP(p) particles in bright field.

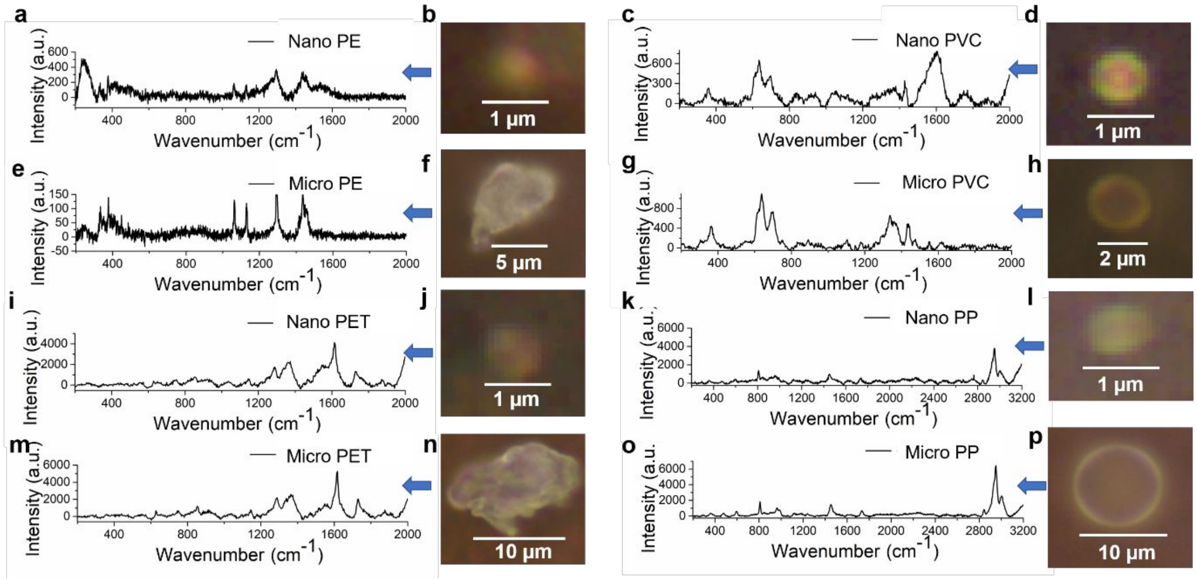

Fig. S4 Quantitative detection of NPs in sea salt by SERS. (a) Raman mapping image of PS in sea salt. (b) Raman spectra of particle identified as PS in the sea salt sample. (c) The reference standard spectra of PS.

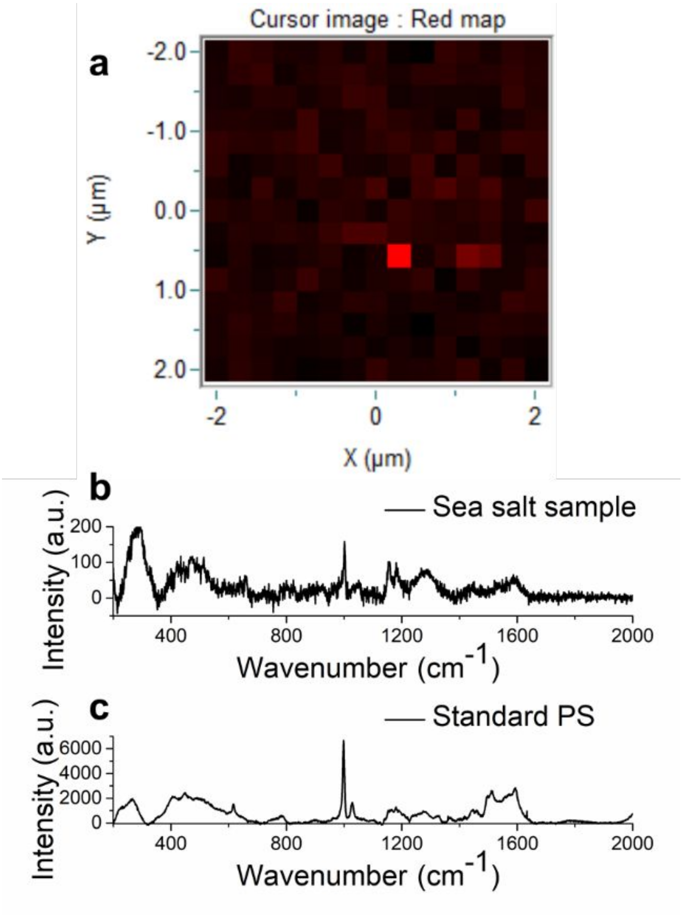

Fig. S5 Nanoparticles containing C, O elements photographed under SEM. The scale bar is 1 μm.

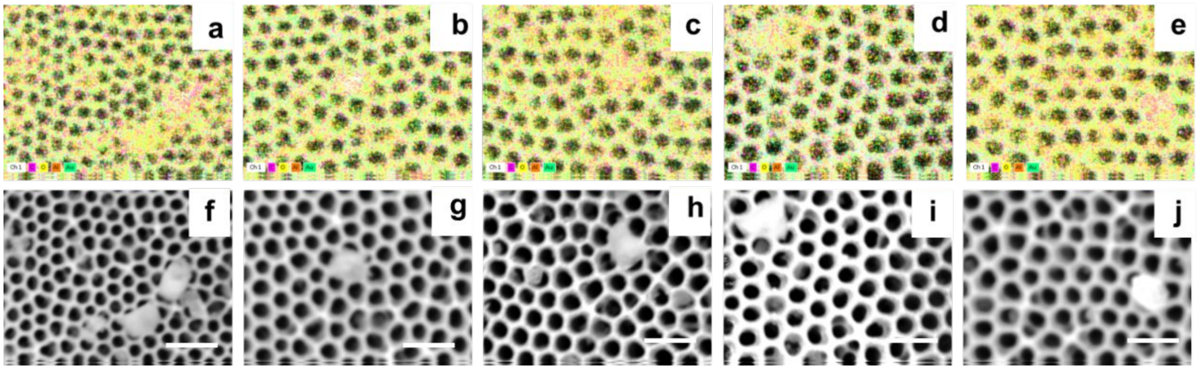

Fig. S6 Spectral decomposition analysis of the SRS spectra. Particle 1 in Fig. S10c identified as PS(a) and PVA(b). The last particle in Fig. S11b identified as PE(c) and PP(d). The first particle in Fig. S11b identified as PP(e). The last particle in Fig. S11c identified as PP(f). Particle 2 and 3 in Fig. 5e identified as PE(g-h).

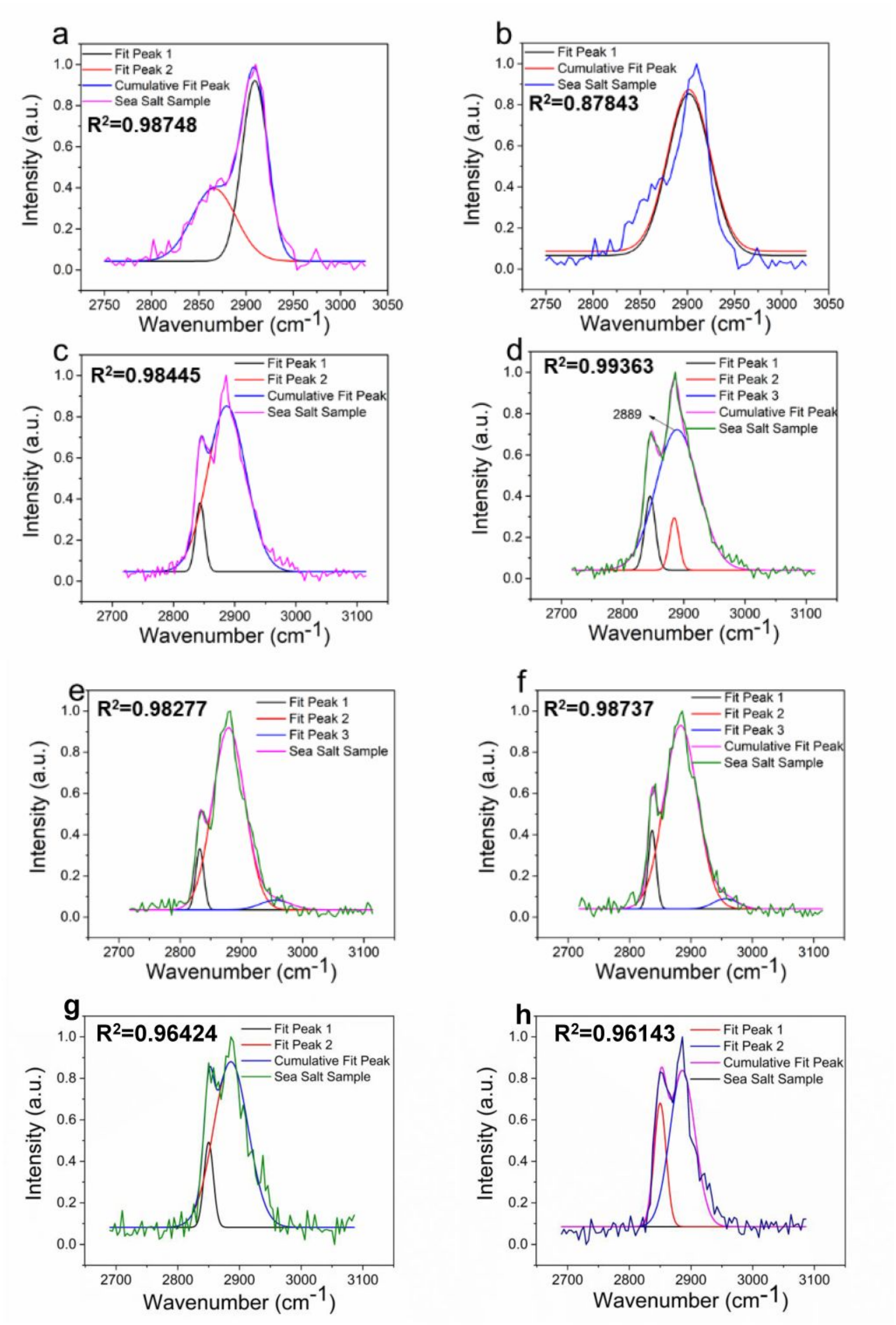

Fig. S7 Spectral decomposition analysis of the SRS spectra of standard PS(a), PE(b), PP(c), PMMA(d), PVA(e), PVC(f).

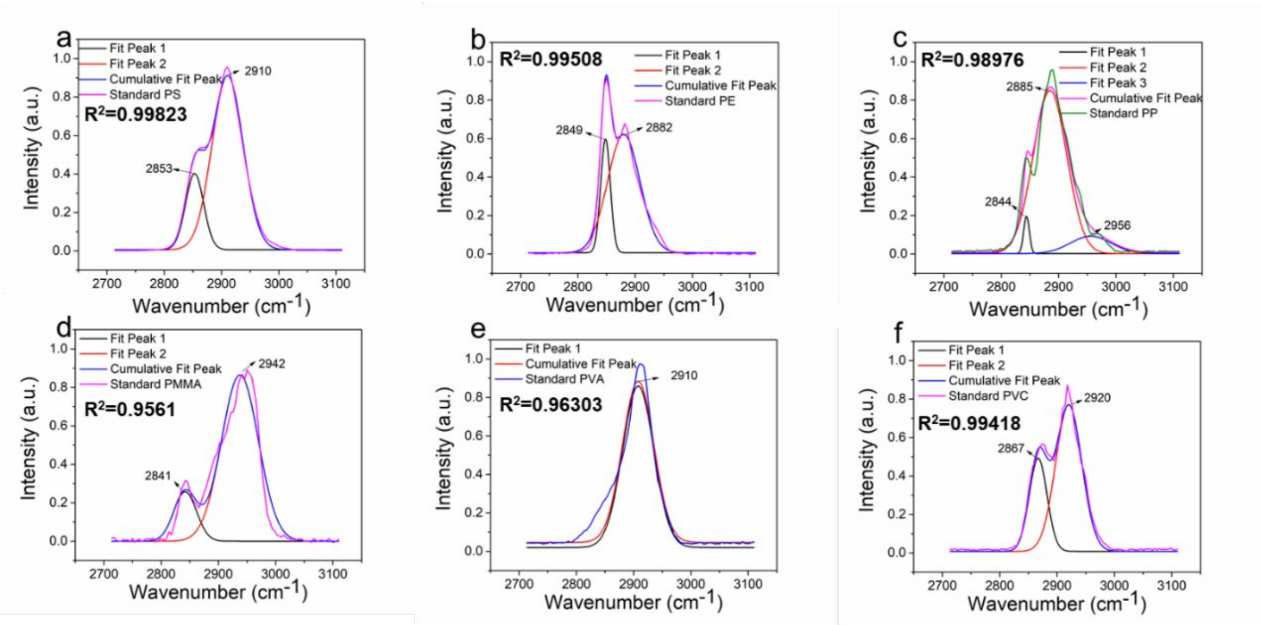

Spectral decomposition analysis of the SRS spectra of standard PS, PE, PP, PMMA, PVA, PVC are shown in Figure 8. For instance, in the case of PS, the 2853 cm^{-1} and 2910 cm^{-1} peaks are attributed to C-H (-CH_2) stretching and C-H (-CH) stretching, respectively [1]. Similarly, for PE, the 2849 cm^{-1} and 2882 cm^{-1} peaks are attributed to C-H (-CH_2) stretching [2], while for PP, the 2844 cm^{-1}, 2885 cm^{-1}, and 2956 cm^{-1} peaks are attributed to C-H (-CH_3) stretching [3]. For PMMA, the 2841, 2942 cm^{-1} are attributed to combination band involving O-CH_3, C-H of O-CH_3 stretching, C-H stretching [4-5]. For PVA, the 2910 cm^{-1} are attributed to C-H (-CH_2) stretching [6]. For PVC, the 2920 cm^{-1} are attributed to C-H (-CH_2) stretching [6].

Fig. S8 SRS imaging and spectra of nano PE detected in sea salt samples from No.3 in the Mediterranean region. a, b, and c are the results obtained from three replicate experiments, respectively.

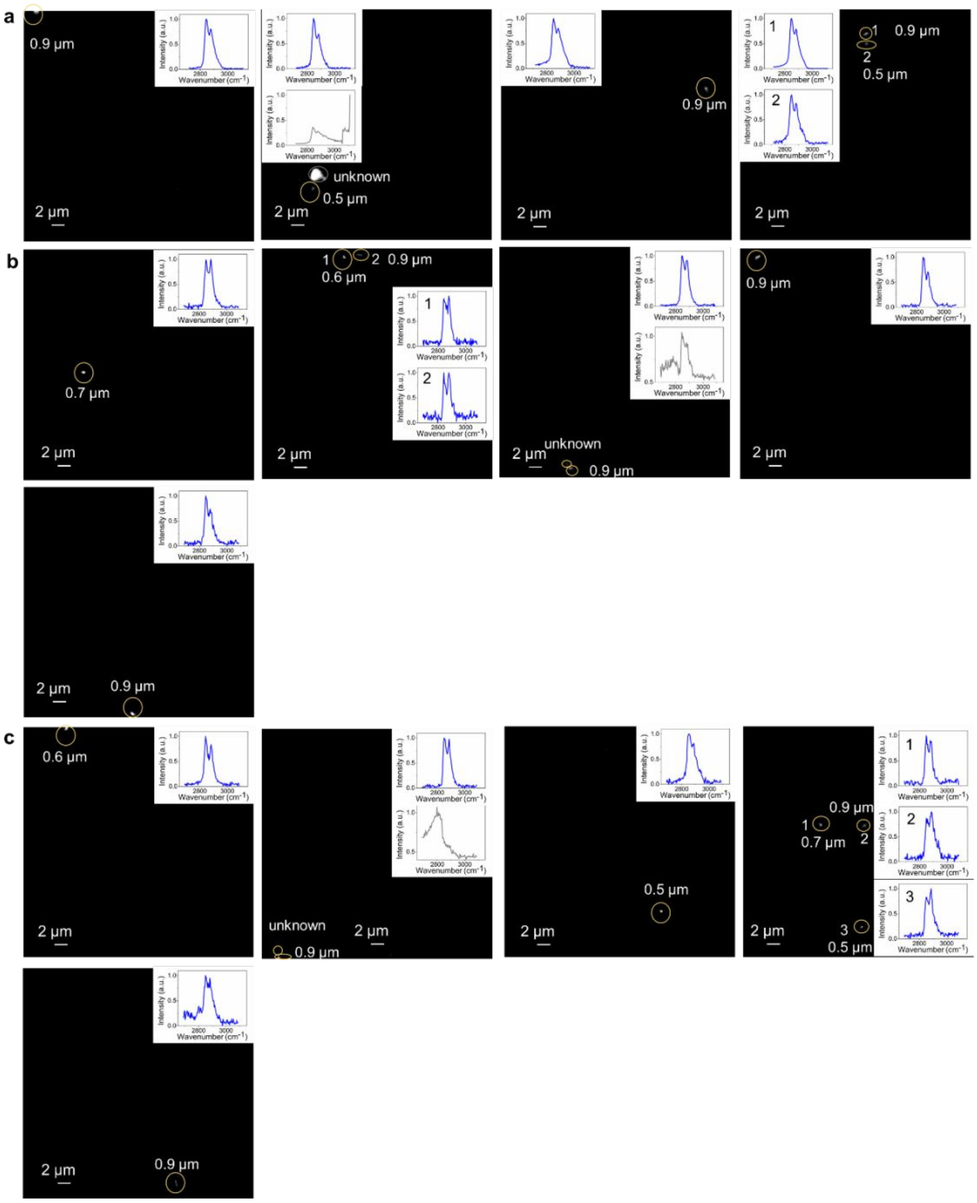

Fig. S9 SRS imaging and spectra of nano PE and PS detected in sea salt samples from No.1 Huai Salt production area in China. a, b, and c are the results obtained from three replicate experiments, respectively.

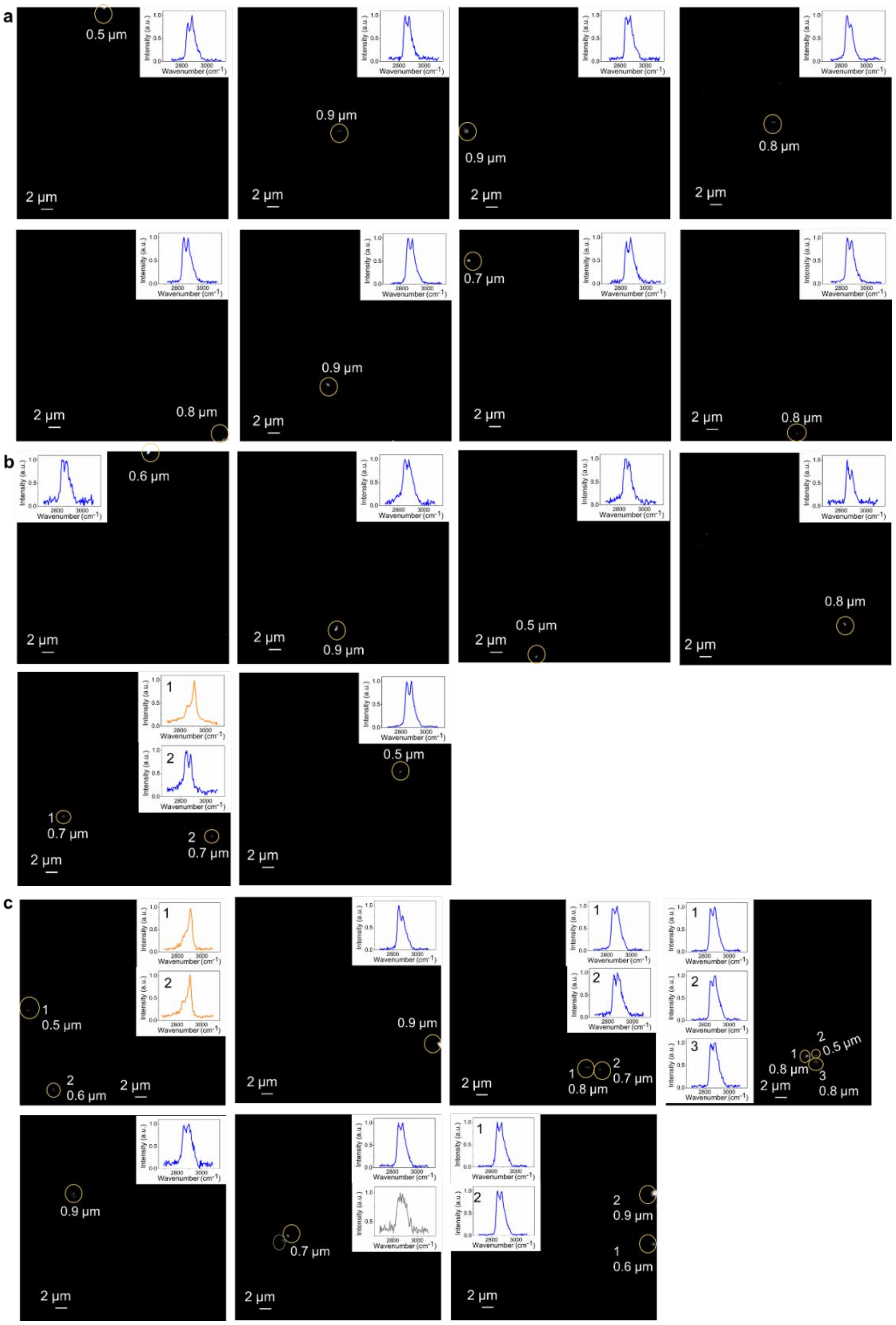

Fig. S10 SRS imaging and spectra of nano PE and PP detected in sea salt samples from No.6 Sinan Sea in Korea. a, b, and c are the results obtained from three replicate experiments, respectively.

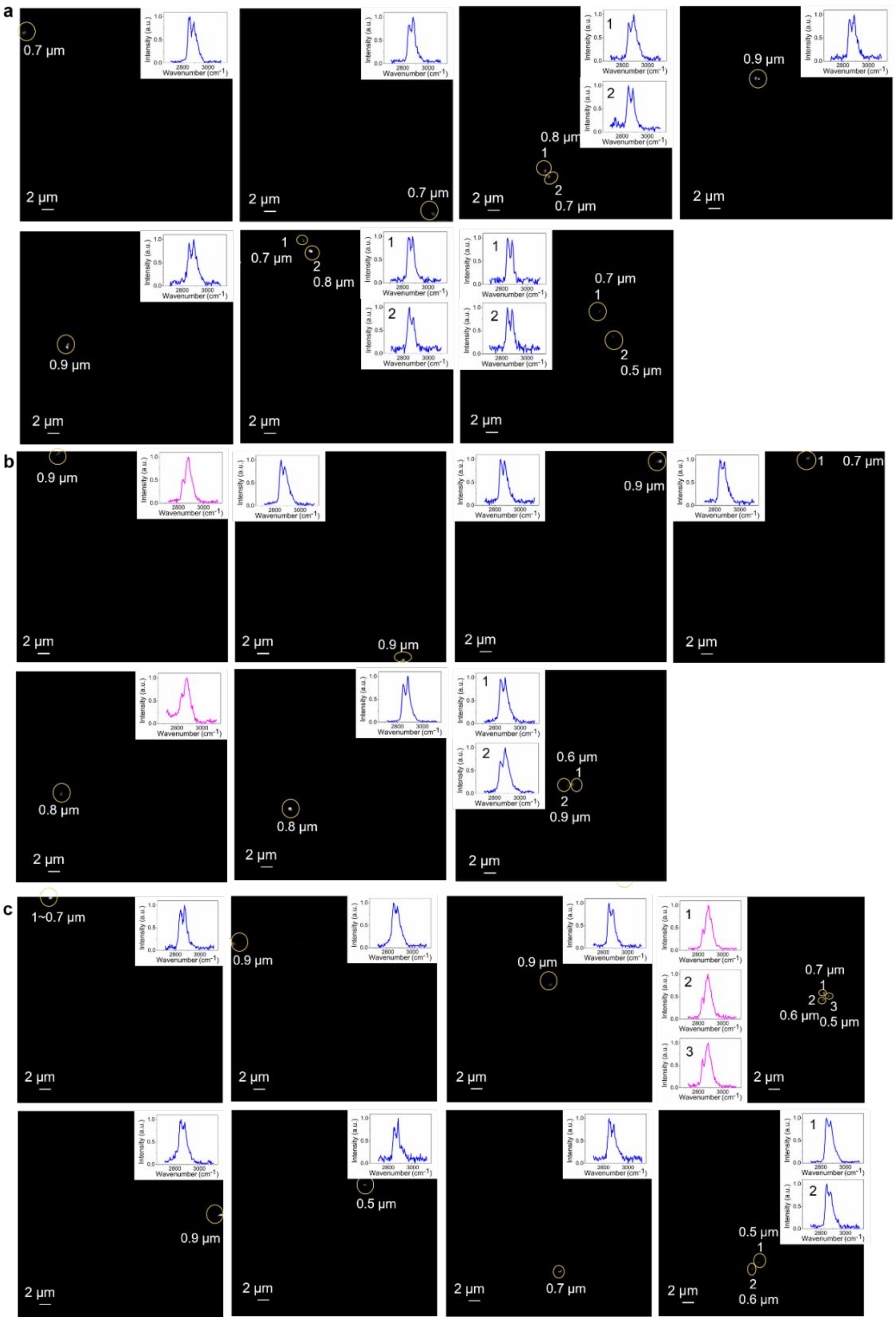

Fig. S11 SRS imaging and spectra of nano PE detected in sea salt samples from No.2 harvested from the frigid current between Australia and Antarctica. a, b, and c are the results obtained from three replicate experiments, respectively.

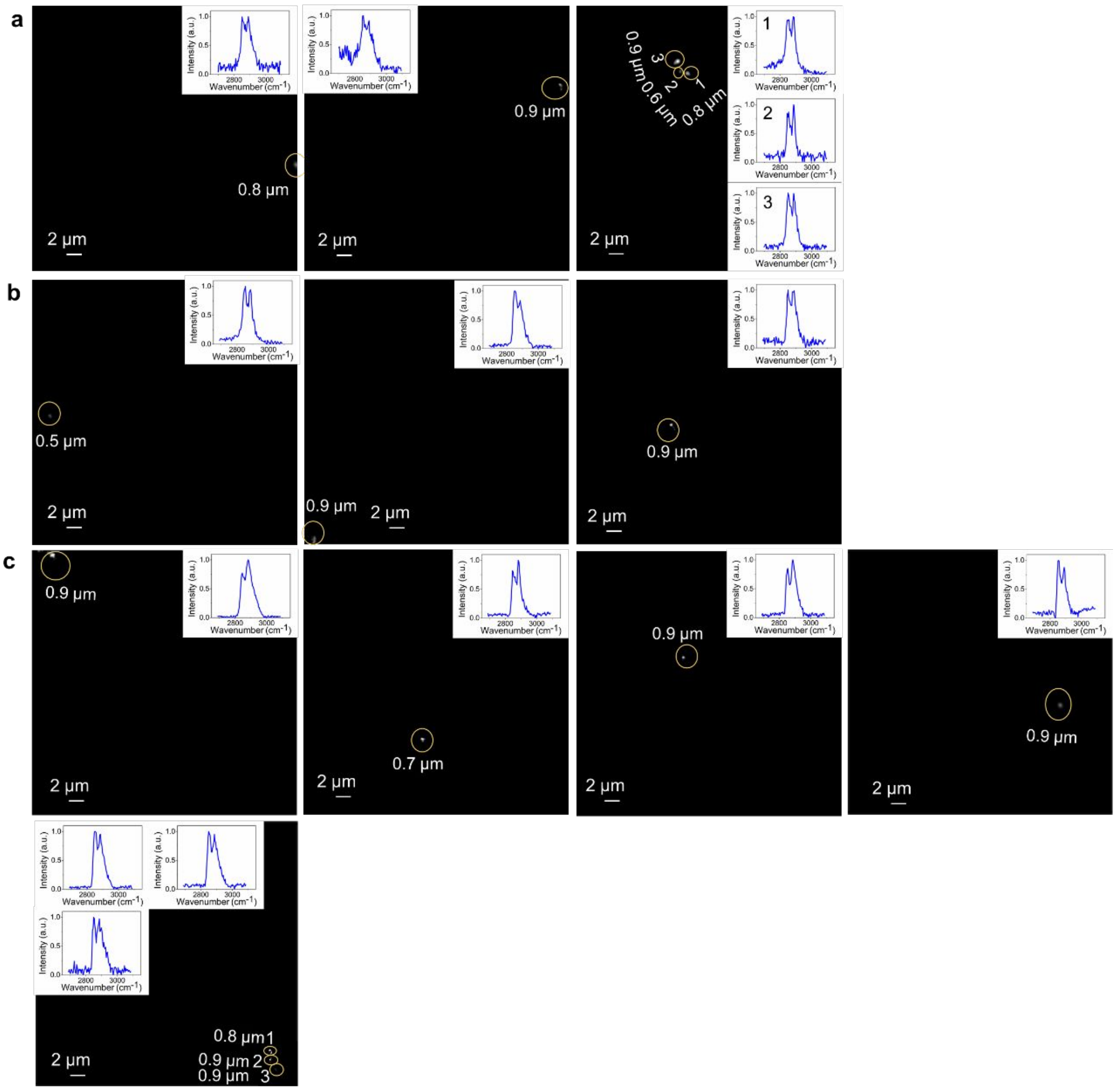

Fig. S12 SRS imaging and spectra of nano PE and PP detected in sea salt samples from No.5 Seto Inland Sea in Japan. a, b, and c are the results obtained from three replicate experiments, respectively.

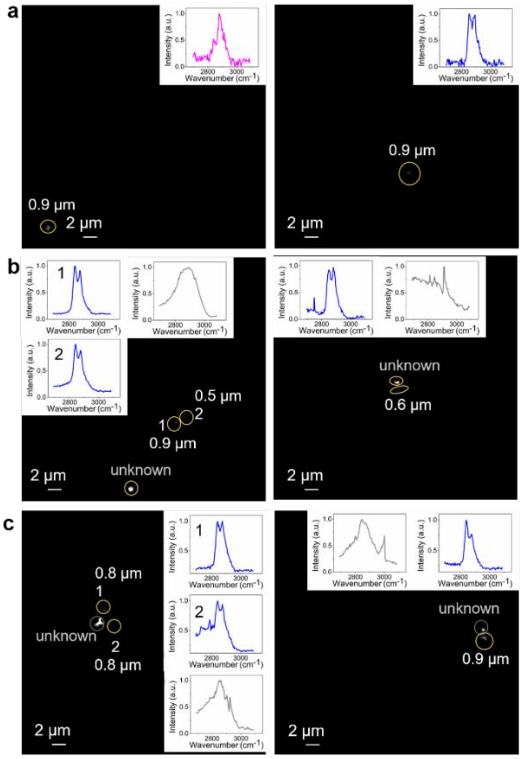

Fig. S13 SRS imaging and spectra of nano PE detected in sea salt samples from No.4 in the French North Atlantic Ocean. a, b, and c are the results obtained from three replicate experiments, respectively.

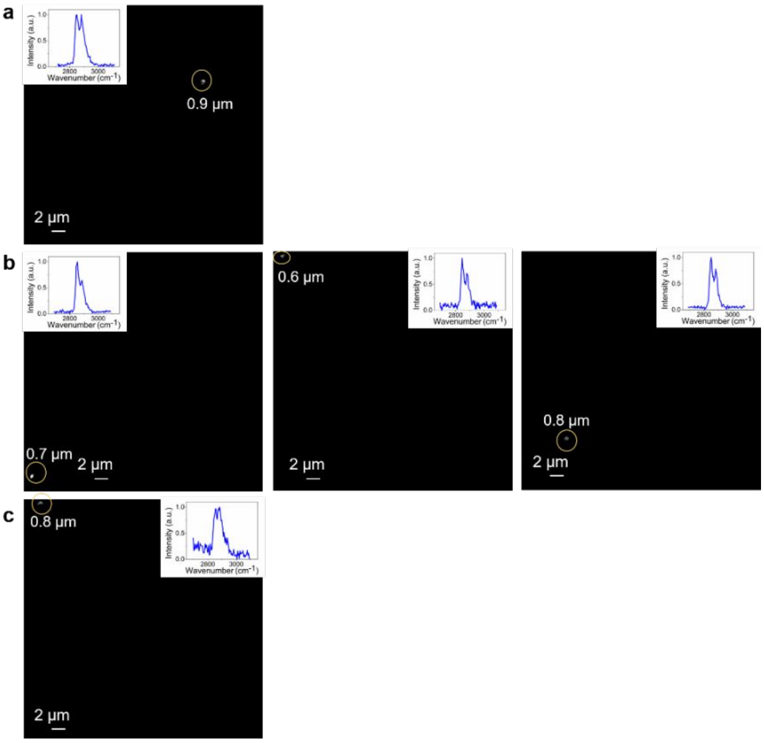

Fig. S14 The amount of nanoplastics contained in 200 g of sea salt.

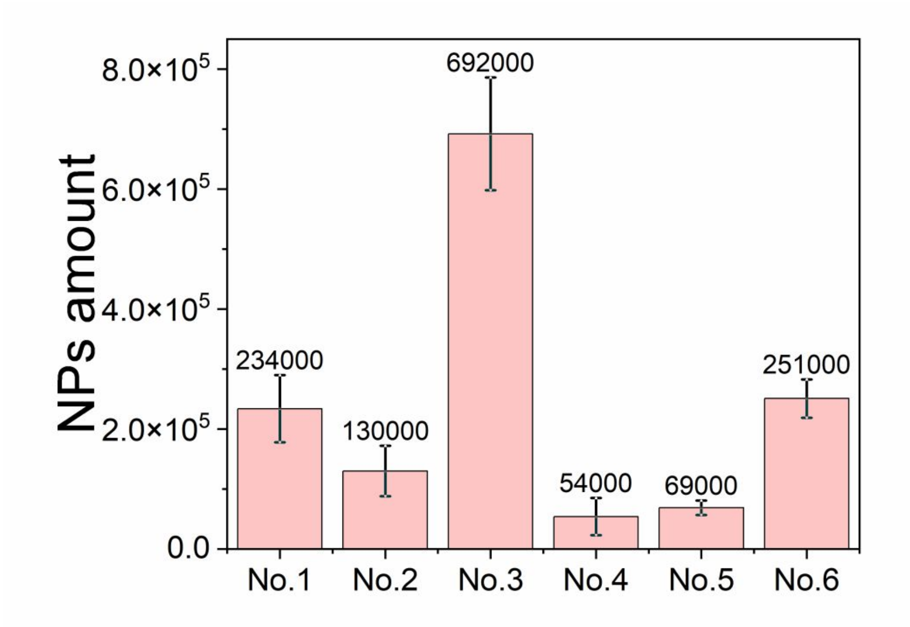

Fig. S15 The locations of 10 test areas on the substrate for SRS study. The size of each square test area is 35.33 μm × 35.33 μm.

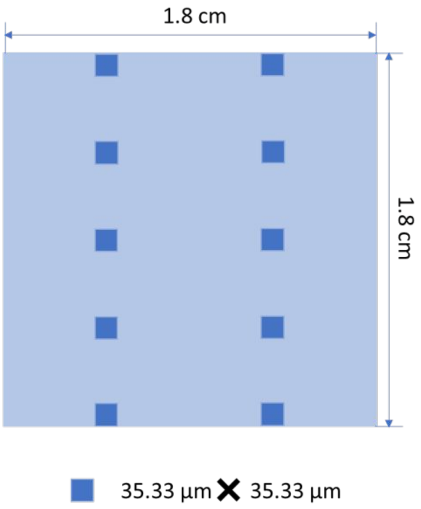

Fig. S16 Imaging of 10 areas of the SRS swept standard sample, a-c are the results of 3 replicates, respectively. The scale bar is 2 μm.

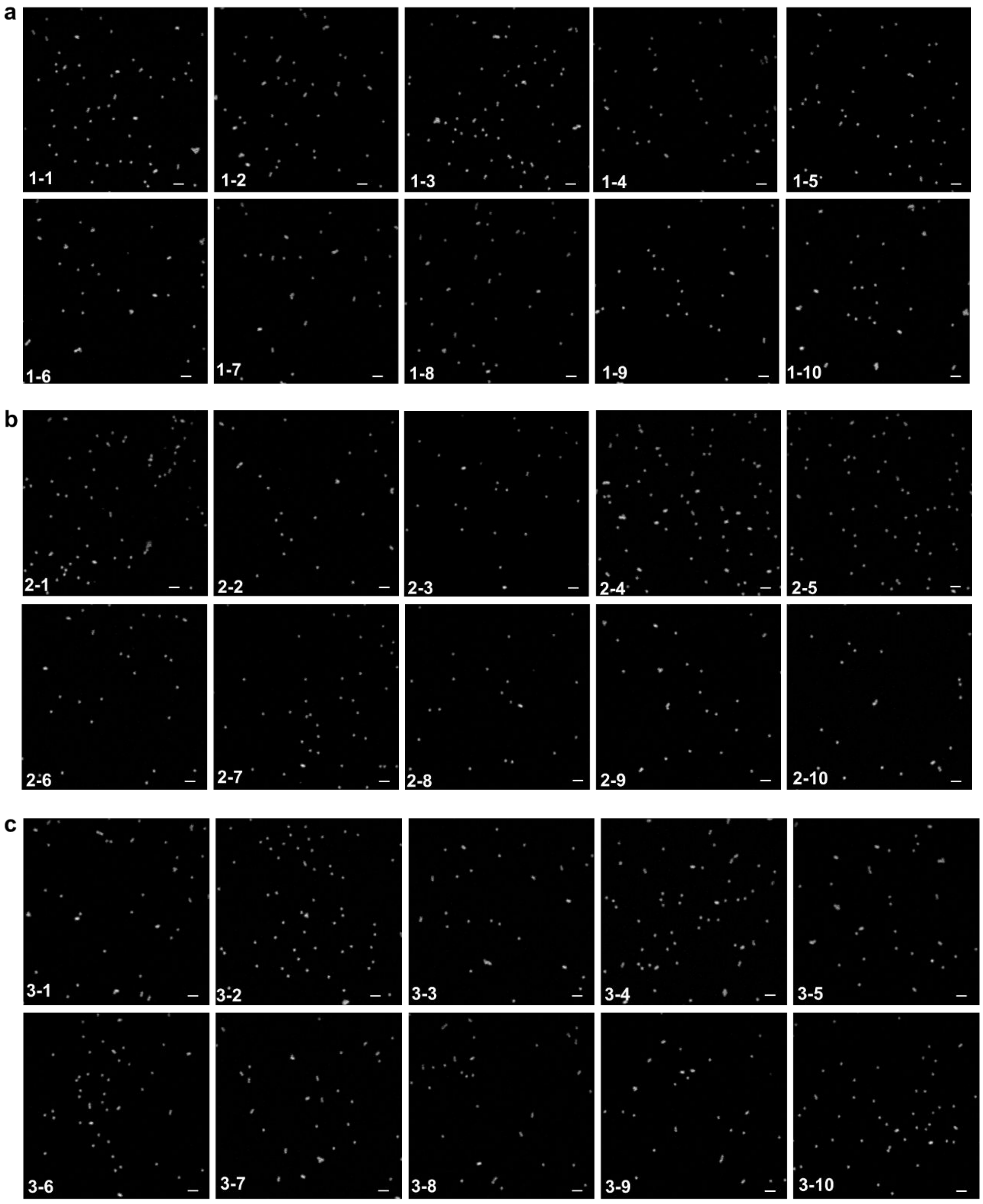

Fig. S17 SRS imaging and spectra of nano PE detected in sea salt samples from No.6 Sinan Sea in Korea. a, b, and c are the results obtained from the experiment was tested 30 areas on one sample, respectively.

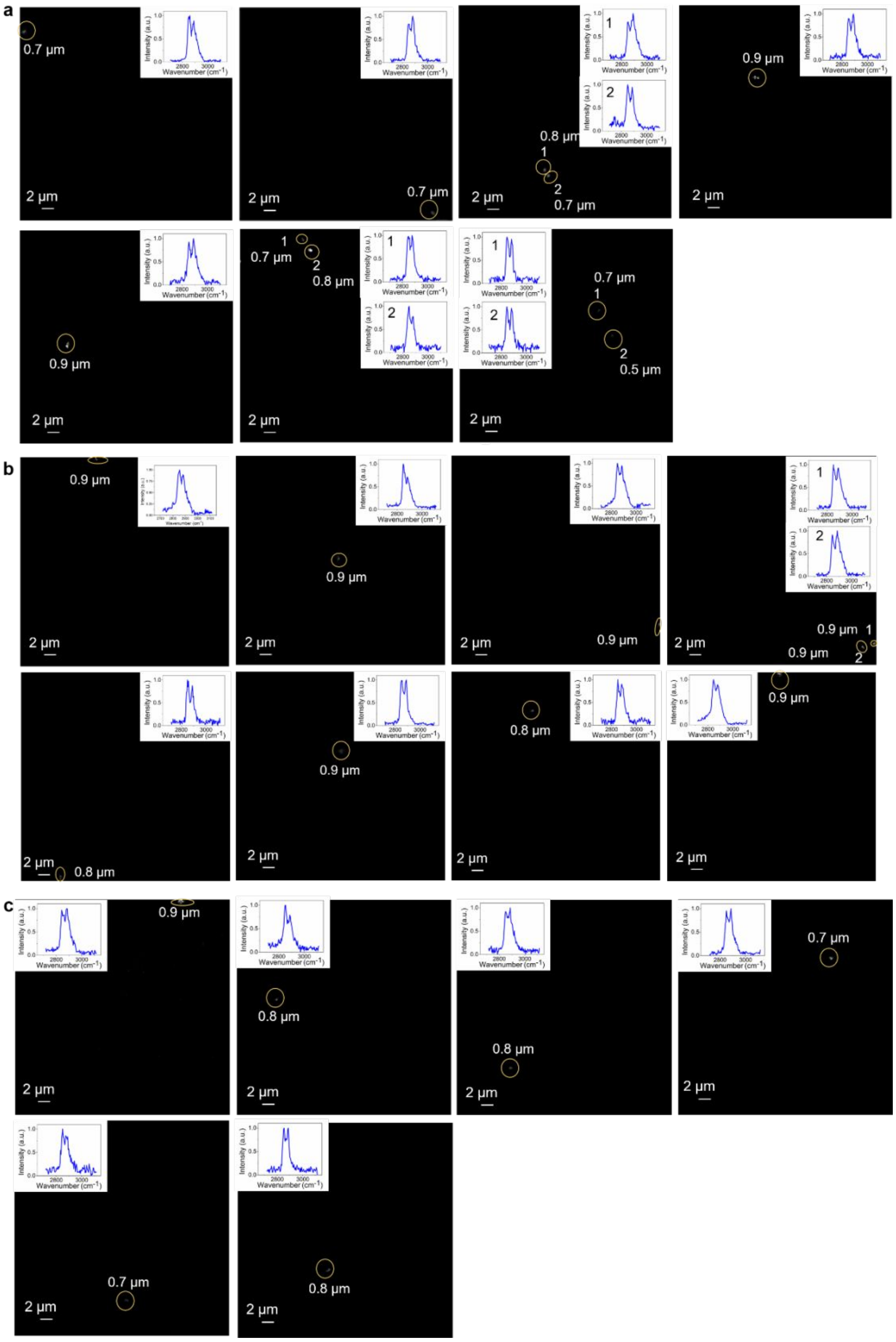

Fig. S18 The locations of 30 test areas on the substrate for SRS study. The size of each square test area is 35.33 μm × 35.33 μm.

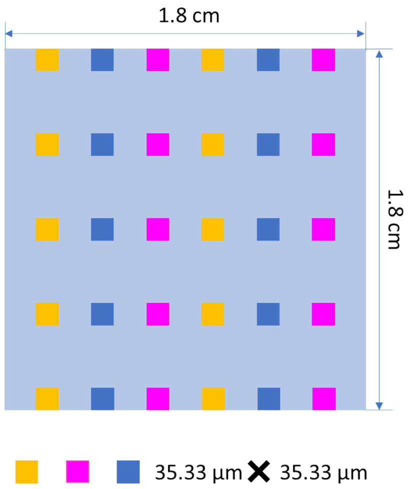

Fig. S19 The amounts of NPs that people in different regions would ingest through sea salt per year. Approximate locations of the sources of the sea salts were marked according to brand labels and other available information.

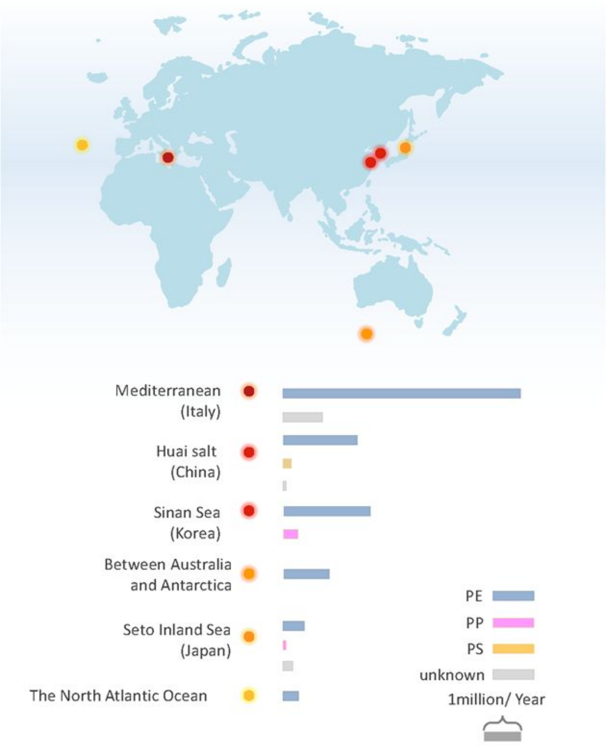

Table S1. Summary of actual sample data of sea salt.

No.	Sea salt weights (a sample)	Region	NPs amount
No 1	200 g	Huai salt production areas of China	234,000±56,000
No 2	200 g	Frigid current between Australia and Antarctica	130,000±42,000
No 3	200 g	Mediterranean Sea	692,000±94,000
No 4	200 g	The North Atlantic Ocean	54,000±31,000
No 5	200 g	Seto Inland Sea, Japan	69,000±12,000
No 6	200 g	Sinan Sea, Korea	251,000±32,000
No 7	0 g	Blank experiment	Not Found

Table S2. SRS quantification results of a known amount of 500 nm PS to verify the accuracy of 10 testing areas method (repeated three times).

Serial number	Number of PS detected	Serial number	Number of PS detected	Serial number	Number of PS detected
1-1	42	2-1	46	3-1	23
1-2	32	2-2	18	3-2	55
1-3	45	2-3	22	3-3	19
1-4	33	2-4	51	3-4	41
1-5	38	2-5	59	3-5	20
1-6	18	2-6	24	3-6	37
1-7	17	2-7	30	3-7	16
1-8	22	2-8	21	3-8	19
1-9	27	2-9	16	3-9	18
1-10	19	2-10	17	3-10	40
Average	29.3		30.4		28.8
Theoretical average	35		35		35
Accuracy	83.7%		86.9%		82.3%

SI References

[1] Sears W M, Hunt J L, Stevens J R. 1981. Raman-scattering from polymerizing styrene .1. vibrational-mode analysis. Journal of Chemical Physics, 75(4): 1589-1598. https://doi.org/10.1063/1.442262.

[2] Gall M J, Willis H A, Cudby M E A, Peacock C J,Hendra P J. 1972. Laser-raman spectrum of polyethylene -assignment of spectrum to fundamental modes of vibration. Spectrochimica Acta Part a-Molecular Spectroscopy, A 28(8): 1485-&. https://doi.org/10.1016/0584-8539(72)80118-1

[3] deBaez M A, Hendra P J, Judkins M. 1995. The Raman spectra of oriented isotactic polypropylene. Spectrochimica Acta Part a-Molecular and Biomolecular Spectroscopy, 51(12): 2117-2124. https://doi.org/10.1016/0584-8539(95)01512-1.

[4] Willis H A, Zichy V J I,Hendra P J. 1969. Laser-raman and infra-red spectra of poly (methyl methacrylate). Polymer, 10(9): 737-&. https://doi.org/10.1016/0032-3861(69)90101-3.

[5] Xu X S, Ming H, Zhang Q J,Zhang Y S. 2002. Properties of Raman spectra and laser-induced birefringence in polymethyl methacrylate optical fibres. Journal of Optics a-Pure and Applied Optics, 4(3): 237-242

[6] Krimm S, Liang C Y. 1956. Infrared spectra of high polymers .4. polyvinyl chloride, polyvinylidene chloride, and copolymers. Journal of Polymer Science, 22(100): 95-112. https://doi.org/10.1002/pol.1956.1202210012.
